# Modeling Phenological and Physiological Responses to Climate Warming in a Hypothetical Migratory Songbird–Mosquito System

**DOI:** 10.1002/ece3.72648

**Published:** 2025-12-11

**Authors:** Isabella G. Ragonese, Sonia Altizer, Courtney C. Murdock, Richard J. Hall

**Affiliations:** ^1^ Odum School of Ecology University of Georgia Athens Georgia USA; ^2^ Center for the Ecology of Infectious Diseases University of Georgia Athens Georgia USA; ^3^ Department of Environmental Conservation University of Massachusetts Amherst Massachusetts USA; ^4^ Department of Entomology University of Georgia Athens Georgia USA; ^5^ Department of Entomology, College of Agriculture and Life Sciences Cornell University Ithaca New York USA; ^6^ Cornell Institute for Host Microbe Interaction and Disease Cornell University Ithaca New York USA; ^7^ Department of Infectious Diseases, College of Veterinary Medicine University of Georgia Athens Georgia USA

**Keywords:** climate change, infectious disease, mathematical model, phenology, physiology

## Abstract

Given the strong temperature dependence of ectothermic vector physiology, climate warming is expected to profoundly impact many vector‐borne diseases. Notably, endothermic hosts can also respond to warming by altering the timing of life history events like seasonal migration and reproduction, but relatively few predictive models of vector‐borne disease have considered both phenological and physiological responses to climate warming. Here, we extend the Ross‐MacDonald model for a vector‐borne disease to incorporate temperature effects on host and vector phenology and physiology. We use this model to understand how projections of moderate and severe warming influence the emergence of a hypothetical vector‐borne disease in a migratory bird. Modeled vector and host infection prevalence always increased under warming, and the increase was amplified when hosts failed to update their arrival phenology to keep pace with breeding site resources. While extreme warming scenarios yielded the highest infection prevalence, reduced vector survival in the hottest months caused late‐season declines in transmission, altering seasonal patterns of infection. By considering host, vector, and parasite responses to temperature together, our modeling framework could be employed to help decipher otherwise non‐intuitive wildlife infection outcomes under current and future climate conditions.

## Graphical abstract

While changes in both phenology and physiology under climate warming are known to influence infection dynamics, surprisingly few studies have explored their interactive effects. We extended the Ross‐MacDonald model for a vector‐borne disease to explore how projections of moderate and severe warming influence the emergence of a hypothetical vector‐borne disease in a migratory bird host. Both shifts in host breeding phenology and warming severity critically affected infection prevalence and consequences for hosts.

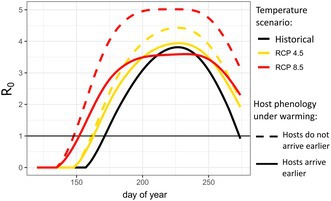

## 1 Introduction

Rising global temperatures are expected to alter species interactions and influence infectious disease transmission, with far‐reaching consequences for wildlife conservation and public health (Altizer et al. 2013; Rohr and Cohen 2020). Many studies have investigated how climate warming affects certain hosts, parasites, or vectors, often focusing on an individual species' physiology, phenology (seasonal timing), behavior, or distribution (Lafferty 2009; Elbers et al. 2015; Levi et al. 2015; Cizauskas et al. 2017; Caminade et al. 2019; Mordecai et al. 2019; Prather et al. 2023). Recent work has started to consider that if host, vector, or parasite species exhibit different physiological, behavioral, and phenological responses to temperature, the strength of their interactions might also change in ways that either exacerbate or reduce transmission (e.g., Gethings et al. 2015; Gehman et al. 2018; Cohen et al. 2019; Peacock et al. 2022). While both changes in phenology and altered physiology under climate warming are known to influence infection dynamics, surprisingly few studies have explored their interactive effects, especially in a multi‐species context.

The physiologies of parasites, ectothermic hosts, and vectors are sensitive to changes in temperature in ways that profoundly influence infection dynamics. Thermal performance curves depict the relationship between temperature and a physiological rate related to an organism's ability to survive, mature, or reproduce (Molnár et al. 2017). These curves are typically hump‐shaped, with focal trait performance peaking at an optimum temperature and falling to zero at temperature extremes (Angilletta 2006). Host, parasite, and vector traits often have distinct thermal performance curves that can vary quantitatively and qualitatively, and in combination, thermal performance curves can provide insight into how interacting species perform relative to each other across temperature (Nowakowski et al. 2016; Cohen et al. 2017). In vector‐borne diseases, many studies have documented the thermal performance of transmission parameters including vector survival, biting rate, and pathogen development within vectors (e.g., Reisen et al. 1992; Paaijmans et al. 2013; Ciota et al. 2014; Paull et al. 2017; Mordecai et al. 2019; Pathak et al. 2019; Shocket et al. 2020). These studies have provided insight into how the transmission potential of pathogens changes seasonally, spatially, and in response to climate warming (LaPointe et al. 2010; Huber et al. 2018; Tesla et al. 2018; Kamiya et al. 2020; Miazgowicz et al. 2020; Villena et al. 2022). While warming climates have generally been predicted to increase parasitism in temperate regions (e.g., Ogden et al. 2008; Paaijmans et al. 2010; Cohen et al. 2020), differential responses of parasites and hosts at warm range edges or thermal extremes can lead to reductions in infection prevalence or even parasite extinction (Lafferty and Mordecai 2016; Gehman et al. 2018; Ryan et al. 2019; Ragonese et al. 2024).

In addition to direct physiological effects, environmental temperature can change the timing and synchrony of key life history events, but the degree to which organisms shift phenology in response to climate warming is not consistent across species (Thackeray et al. 2016; Kronfeld‐Schor et al. 2017; Robertson et al. 2024; Belitz et al. 2025). Phenological mismatch occurs when the seasonal timing of key events (such as migration or invertebrate emergence) diverges for interacting species under environmental change, altering their temporal overlap or the nature of their interaction. When phenological mismatch occurs between a consumer and its resource, the consumer can experience reduced reproductive success (Both et al. 2006; Saino et al. 2011; Visser and Gienapp 2019). As hosts, parasites, and vectors respond to climate change, differential phenological responses can impact host–parasite contact rates, shaping transmission potential and infection outcomes (Altizer et al. 2013; Reece et al. 2017; MacDonald et al. 2021; McDevitt‐Galles et al. 2024). Earlier breeding onset in songbirds, for instance, has been associated with either increasing or decreasing synchronicity with vector and ectoparasite emergence (Møller 2010). These phenological mismatches have been shown to result in substantial declines in parasite transmission, particularly in systems in which portions of the parasite life cycle are sensitive to temperature variation (e.g., ectothermic intermediate hosts (Paull and Johnson 2014) or parasite stages that develop in the environment (Gethings et al. 2015)).

Mathematical models are useful tools for examining how multiple species' responses to warming impact infectious disease. Previous models have leveraged our understanding of temperature effects on physiology to predict transmission under current temperature and future warming scenarios (e.g., Li et al. 2016; Ryan et al. 2019; Endo and Amarasekare 2022). Other models have explored how phenological responses to warming result in changes in host fitness and disease transmission (e.g., Brown and Rohani 2012; Murdock et al. 2013; Hall et al. 2016). A recent model of a parasitic nematode infecting migratory caribou incorporated climate impacts on parasite thermal performance and behavior in migratory versus sedentary hosts (Peacock et al. 2022). Their model allowed them to assess how climate warming could impact parasite physiology in addition to spatiotemporal overlap between parasites and hosts (Peacock et al. 2022). To date, however, few models of vector‐borne disease have combined climate‐induced shifts in host and vector phenology with physiological responses to higher temperatures.

Here we develop a modeling framework for the dynamics of a hypothetical emerging vector‐borne disease under climate warming that integrates the phenological responses of the migratory host and vector with the physiological responses of the vector and pathogen. Given that vectors are ectothermic, vector phenology is likely more sensitive to changes in breeding site temperature relative to migratory bird phenology (Bolling et al. 2007; Prather et al. 2023). Thus, we assume that under warming, vector emergence always shifts earlier relative to the historical scenario, and physiological rates always respond to daily temperature. We explore two possibilities for how host phenology responds to climate warming: (1) no advancement in host migratory timing, leading to a mismatch with resources that lowers host reproductive success (Ambrosini et al. 2011; Saino et al. 2011; Shaftel et al. 2021; Youngflesh et al. 2023); and (2) advanced host arrival under warming. We predicted that both physiological and phenological responses would determine infection outcomes. While warming‐induced phenological shifts could alter seasonal overlap between hosts and vectors, temperature‐dependent physiological responses could alter vectorial capacity through changes to vector‐host ratios, biting rates, and competence for infection (Smith et al. 2012).

## 2 Model and Methods

### 2.1 Overview

We use differential equations to track the population and infection dynamics of a migratory avian host and mosquito vector over a single host breeding season, following the first introduction of a novel pathogen (i.e., assuming no pre‐existing immunity in the host). We incorporate climate warming impacts on host and vector phenology and vital rates, assessing outcomes under pre‐warming, moderate, and severe warming scenarios. Warming affects vector emergence timing and, in some scenarios, host arrival date (which in turn influences reproductive success). Temperature dependence in transmission traits is incorporated by linking vector mortality rate, biting rate, and pathogen development rate to daily temperature values. For tractability, we assume that only the focal host species can acquire and transmit the pathogen and that mosquitoes preferentially bite this host, as has been assumed in past models of avian vector‐borne diseases (Wonham et al. 2004; Bowman et al. 2005; Cruz‐Pacheco et al. 2005; Murdock et al. 2013; Brown and Hall 2018; Becker et al. 2020; Owen et al. 2021; Fesce et al. 2023). We acknowledge that this assumption omits some of the real‐world complexity of multi‐host vector‐borne disease transmission, where hosts can show marked variability in competence for infection and migratory propensity (Table S2). Given our goal of demonstrating theoretically that both phenological and physiological responses can shape emerging infection dynamics, a simpler, single‐host model structure was warranted.

We parameterized our model with data on West Nile Virus (WNV), *Culex* mosquitoes, and migratory American robins ( *Turdus migratorius* ), an important host implicated in maintaining WNV circulation (Kilpatrick, Daszak, et al. 2006; Kilpatrick, Kramer, et al. 2006; Hamer et al. 2009; Simpson et al. 2012). We chose this system because the thermal performance of *Culex* mosquito and WNV traits has been well‐quantified (Reisen et al. 2006; Ciota et al. 2014; Paull et al. 2017; Shocket et al. 2020), and because data on robin fecundity and survival, and temperature cues for migratory arrival (Bent 1949) are readily available. We stress that the goal of our study is not to make quantitative forecasts about WNV dynamics, as this would potentially involve modeling multiple host and vector species. Rather, we modify a simple single‐host, single‐vector model to investigate the qualitative effects of simultaneous phenological and physiological responses to warming. We use the model to explore how novel pathogen emergence could be shaped by distinct host and vector responses.

### 2.2 Model Structure

**FIGURE 1:**
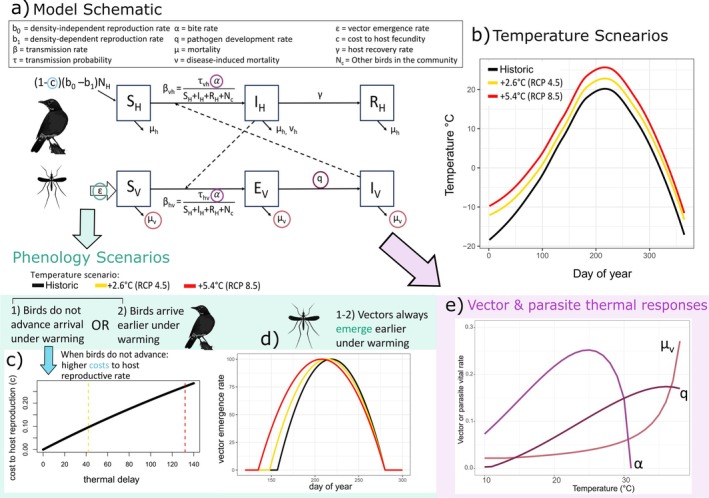
Schematic diagram depicting the model compartments and parameters that determine the transitions of individuals (a). Temperature scenarios across 1 year (b). Phenology scenarios for avian host and mosquito vectors, including the cost to host reproduction when they do not advance arrival (c) and the annual vector emergence curves for all three temperature scenarios (d). Thermal responses of vital rates (e), biting (*α*), vector mortality (*μ*_v_), and pathogen development rate (*q*).

The model is a modification of the Ross‐MacDonald model for vector‐borne pathogen transmission (Macdonald 1957; Smith et al. 2012), tracking the status of susceptible (*S*_*H*_), infected (*I*_*H*_), and recovered (*R*_*H*_) hosts, and susceptible (*S*_*V*_), exposed (*E*_*V*_), and infected (*I*_*V*_) vectors (Figure 1a):

(1a) $$\frac{dS_{H}}{\mathrm{dt}}=\left(1-c\right)\left(b_{0}-b_{1}N_{H}\right)N_{\text{breed}}-\mu_{h}S_{H}-\frac{\beta_{\mathrm{vh}}S_{H}I_{V}}{N_{H}+N_{C}}$$

(1b) $$\frac{dI_{H}}{\mathrm{dt}}=\frac{\beta_{\mathrm{vh}}S_{H}I_{V}}{N_{H}+N_{C}}-\left(\mu_{h}+\nu_{h}+\gamma \right)I_{H}$$

(1c) $$\frac{dR_{H}}{\mathrm{dt}}=\gamma I_{H}-\mu_{h}R_{H}$$

(1d) $$\frac{dS_{V}}{\mathrm{dt}}=\varepsilon -\mu_{v}S_{V}-\frac{\beta_{\mathrm{hv}}I_{H}S_{V}}{N_{H}+N_{C}}$$

(1e) $$\frac{dE_{V}}{\mathrm{dt}}=\frac{\beta_{\mathrm{hv}}I_{H}S_{V}}{N_{H}+N_{C}}-\left(\mu_{v}+q\right)E_{V}$$

(1f) $$\frac{dI_{V}}{\mathrm{dt}}=qE_{V}-\mu_{v}I_{V}$$

The sizes of the host and adult vector populations are denoted by *N*_*H*_ (= *S_H_* + *I_H_* + *R_H_*) and *N*_*V*_ (= *S_V_* + *E_V_* + *I_V_*) respectively, while *N*_breed_ is the number of reproductive adult birds returning to the breeding site each year adjusted by the breeding season per capita mortality rate (see Appendix S1). *N*_*C*_ is the number of other, non‐competent bird hosts in the community, which can dilute vector bites (set to 0 in the main model, but see Appendix S10). Birds have a per capita reproduction rate *b*_0_—*b*_1_ *N*_*H*_, where *b*_0_ is the maximum reproduction rate and *b*_1_ scales how much the reproduction rate decreases with bird density. We assume that, due to a mismatch with food resources, host reproduction declines under warming if birds do not advance their arrival date (e.g., Both et al. 2006; Jones and Cresswell 2010; Saino et al. 2011; Ross et al. 2017). This reduction is modeled as a cost, *c*, to reproduction (see Section 2.4, Figure 1c). Juveniles recruit to the population after a delay (*b_delay_* days after arrival) for territory selection, mate finding, and nesting until the end of the breeding season. We assume a constant per capita host mortality rate during the breeding season, *μ*_ℎ_. We also assume host individuals have synchronized arrival (*h*_start_) and departure (*h*_end_) from the breeding site. While age and sex differences can lead to staggered host arrival in some species (Table S2), the simplifying assumption of arrival synchrony allows us to treat host arrival date as a single input variable in our modeling scenarios, making visualization of our results more tractable. Since a study across multiple species found no overall effect of warming on autumn phenology, with variable directional responses among bird species (Van Buskirk et al. 2009), we opt to keep *h*_end_ constant and focus on shifts in arrival phenology.

For simplicity, and because adult mosquito emergence is dependent on factors other than temperature (Rakotoarinia et al. 2022), the seasonal vector emergence rate, *ε*(*t*), is described by a unimodal, quadratic function with peak emergence *ε*_max_ in the middle of the vector activity season (following Brown and Hall 2018) (Figure 1d):

(2) $$\varepsilon \left(t\right)=\frac{4\varepsilon_{\max}}{{\left(v_{\mathrm{end}}-v_{\text{start}}\right)}^{2}}\left(v_{\mathrm{end}}-t\right)\left(t-v_{\text{start}}\right),$$

where *t* is the daily timestep, *v*_start_ is the first day of vector emergence and *v*_end_ is the final day of seasonal vector activity. Vectors experience per capita mortality at rate, *μ*_*v*_, which varies with temperature (see Section 2.4). Given the degree day accumulation needed to cue emergence in our model, vectors always emerge after host arrival, even under warming. We assume that vector survival at lower host densities is not blood meal‐limited, but acknowledge that in some cases, host availability and temperature can jointly influence vector‐borne disease transmission (Dahlin et al. 2024) (Table S2).

The transmission rate from an infected vector to a susceptible host (*β*_*vh*_) is equal to the product of the host's infection probability given an infectious mosquito bite (*τ*_*vh*_) and the vector biting rate (*α*), which depends on temperature (details in Section 2.4). The transmission rate from an infected host to a susceptible vector (*β*_*hv*_) is equal to the product of the infection probability for a susceptible vector given a bite on an infected host (*τ*_*hv*_) and the vector biting rate (*α*). Vectors move from the exposed class to the infected class at a temperature‐dependent rate *q* (see Section 2.4). We assume the vector incurs no costs of infection, but hosts experience disease‐induced mortality at a per capita rate *ν*_*h*_ (as in other models (Rubel et al. 2008; Bergsman et al. 2016; Bhowmick et al. 2020)). Although empirical studies have shown no or low WNV‐induced mortality in American robins (Komar et al. 2003; VanDalen et al. 2013), disease severity varies widely across avian hosts (Marfin et al. 2001; Kilpatrick et al. 2007), and disease‐induced mortality can be especially high for emerging pathogens (Williams et al. 2002; LaDeau et al. 2007; Foppa et al. 2011). Hosts recover from infection at a rate *γ*, and we assume that immunity lasts for the rest of the season (Reisen 1995; Reisen et al. 2006; Rubel et al. 2008; Laperriere et al. 2011).

### 2.3 Climate Data

Our historical and projected temperatures over an annual cycle are derived from a downscaled CNRM_CM5 global climate model for Minnesota, a state within the breeding range of migratory American robins (Vanderhoff et al. 2016). The CNRM_CM5 model simulates past and projected future climate under assumed greenhouse gas emission scenarios (representative concentration pathways, RCPs) (IPCC 2021; Liess et al. 2022). This particular model is one of several available to project Minnesota temperatures (Liess et al. 2022), and we select it because the RCP scenarios resulted in distinct warming levels relative to historical temperature data (Figure 1b). We use CNRM_CM5 to demonstrate that spatially relevant climate data can be incorporated into our framework, but specific climate model choice is unlikely to influence the qualitative interpretation of our results. Our three temperature scenarios are (1) historical baseline temperatures (1980–1999), (2) moderate warming (RCP 4.5 2080–2099; on average + 2.6°C in the bird breeding season), and (3) severe warming (RCP 8.5 2080–2099; on average + 5.4°C in the breeding season) (Figure 1b). The climate model provides us with daily mean temperatures over 1 year for each scenario. We applied the “stats::loess” function to the raw temperature vectors to smooth them and used the smoothed vectors as model inputs. With the *pollen* package in R (v 4.0.5) (Nowosad 2021; RCoreTeam 2022), we also used the temperature vectors to calculate spring accumulated temperature, or degree days (the sum of degrees above 0°C since January 1) using the “gdd” function.

### 2.4 Model Parameterization

Our model is parameterized using data on the American robin ( *Turdus migratorius* ), *Culex* sp. mosquito vectors, and West Nile Virus (WNV). American robins are an abundant, migratory species that have been implicated as important hosts in the transmission of emerging pathogens of human or livestock concern, including WNV (Kilpatrick, Daszak, et al. 2006; Kilpatrick, Kramer, et al. 2006; Molaei et al. 2006; Hamer et al. 2009; Simpson et al. 2012), and Eastern Equine Encephalitis Virus (Armstrong and Andreadis 2022). Estimates of robin fecundity and survival are described in Appendix S1 (DeSante et al. 2015; Brown and Miller 2016; Vanderhoff et al. 2016). Environmental cues like precipitation, temperature, and snowmelt can influence robin spring arrival (Travers et al. 2015; Oliver et al. 2020; Prather et al. 2023). As our model focuses on temperature, we assume that, in scenarios where robin phenology can respond to warming, the migrating birds can track the 2.8°C isotherm (as suggested in Bent 1949). Therefore, their new arrival date is the first day that daily temperature surpasses 2.8°C. We model the cost parameter, *c*, as a proportionate reduction in the reproduction rate that increases with the “thermal delay.” Thermal delay is defined as the difference between degree days on the host arrival date under warming (dd_j_) and degree days on the historical arrival date (dd_opt_); (dd_j_−dd_opt_) (Ambrosini et al. 2011). Under warming, hosts that arrive on their historical date experience higher degree days and are “thermally delayed” relative to spring phenological conditions at the breeding site (Saino et al. 2011) (see Appendix S3). Specifically, the reproduction cost takes the form:

(3) $$c=1-e^{\left(-f_{c}\cdot {\text{t}}_{\text{delay}}\right)},$$

where *t_delay_* (= dd_j_−dd_opt_) is the thermal delay and *f_c_* is a constant controlling the strength of the relationship (Figure 1c; Figure S1).

For the vectors, temperature‐dependent parameters are based on *Culex* mosquito functional forms presented in Shocket et al. (2020) (Figure 1e). The first day adult vectors emerge is dictated by a degree day cue (Bolling et al. 2007; Diuk‐Wasser et al. 2010), and, due to the influence of photoperiod on vector diapause, the last day of vector activity is set at *t =* 280 (~October 7th) for each scenario (Field et al. 2022). Vector mortality rate, *μ*_*v*_, is calculated as the inverse of adult lifespan, which has a linear, mechanistic link to daily temperature (Reisen 1995; Ciota et al. 2014; Shocket et al. 2020). Biting rate is mechanistically linked to daily temperature with a Briѐre function (Reisen et al. 1992; Shocket et al. 2020). We also model the temperature dependence of infection latency by linking pathogen development rate, *q*, to temperature with a Briѐre function (Reisen et al. 2006; Kilpatrick et al. 2008; Shocket et al. 2020). We provide default parameter values in Table 1 and details on the derivation of values in Appendix S2 (Table S1).

**TABLE 1:** Parameter values (and ranges for sensitivity analyses in parentheses), with parameters that depend on degree day, time, or temperature presented in bold as *f* *(dd)*, *f* *(t* *)*, and *f* *(T)*, respectively.

Parameter	Value (day ^−1^)	Definition	Source
Birds			
*h*_start_	***f(T)***	Date of host arrival at breeding site	Bent (1949)
*h*_end_	274	Date of host departure from breeding site	
F_max_	4.5	Maximum fecundity	Brown and Hall (2018), Vanderhoff et al. (2016)
K	1000	Carrying capacity	
*b*_0_	Log(F_max_)/(*h*_end_−*h*_start_)	Max per capita reproductive rate	Brown and Hall (2018)
*b*_1_	*b*_0_/K	Density‐dependent reproductive rate	Brown and Hall (2018)
*b_delay_*	14	Days between host arrival and hatching of new hosts	Vanderhoff et al. (2016)
*c*	***f(dd)***	Cost to the host reproductive rate	Ambrosini et al. (2011), Youngflesh et al. (2023)
f_c_	0.0012 (0–0.0024)	Reproductive cost scaler	
*μ*_h_	0.0002	Per capita mortality	Brown and Hall (2018), DeSante et al. (2015)
*ν*_h_	0.055	Disease‐induced mortality	Rubel et al. (2008), Bergsman et al. (2016)
*μ*_h,nb_	0.003	Non‐breeding mortality	Brown and Hall (2018)
*N*_c_	0 (0–800)	Number of non‐focal birds in the community (bite dilution)	
Vectors			
*v*_start_	***f(dd)***	First day of vector emergence at breeding site	Bolling et al. (2007)
*v*_end_	280	Last day of vector biting activity at breeding site	Diuk‐Wasser et al. (2010), Field et al. (2022)
*ε*_max_	100	Maximum vector emergence rate	Hall et al. (2016), Brown and Hall (2018)
*ε*	*f(t)*	Vector emergence rate	
θ	0 (− 4–4)	*ε*_max_ scaler under warming	
*μ*_v_	***f(T)***	Vector mortality	Shocket et al. (2020)
*α*	***f(T)***	Vector biting rate	Shocket et al. (2020)
Infection			
*τ*_hv_	0.65	Probability of infection (host to vector)	Rubel et al. (2008)
*τ*_vh_	0.32	Probability of infection (vector to host)	Rubel et al. (2008)
*γ*	0.1	Host recovery rate	Rubel et al. (2008), Laperriere et al. (2011)
*q*	***f(T)***	Pathogen development rate	Shocket et al. (2020)

### 2.5 Model Analysis

Model simulations were implemented in RStudio (v 4.0.5) using the differential equation solver *deSolve* (Soetaert et al. 2010). We consider the dynamics of infection in the year of pathogen introduction (i.e., assuming no prior exposure or immunity of hosts). To determine the initial number of hosts returning to breed (*N_breed_*), we solved the disease‐free host model until the host population converged into repeating annual cycles (30 years). We ran the model for five different scenarios, comparing baseline, pre‐warming dynamics to a crossed design of warming (moderate or severe) and host phenology (no advance in host arrival versus advanced host arrival that perfectly tracks temperatures associated with historical arrival). Although many migrants track climate warming imperfectly (i.e., they arrive earlier at breeding grounds but not early enough to track breeding site phenology), we chose to illustrate the two most extreme scenarios of no adaptation versus perfect adaptation for visual clarity, and because preliminary simulations showed that model outcomes for imperfect climate tracking always lay between these two extreme scenarios. Our five scenarios are:

1. historical temperatures and baseline host and vector phenology,
2. moderate warming, hosts arrive on the historical date (i.e., no advance in host phenology), and advanced vector emergence,
3. severe warming, historical host arrival, and advanced vector emergence,
4. moderate warming, earlier host arrival, and advanced vector emergence.
5. severe warming, earlier host arrival, and advanced vector emergence.

### 2.6 Model Outcomes

#### 2.6.1 Transmission Potential

Using the daily disease‐free host and vector abundances, we calculated a metric of transmission potential for each day under each scenario. The daily basic reproductive number, *R*_0_(*t*), is the number of new cases generated by a single infectious host or mosquito introduced into a fully susceptible population arriving on day *t*. We used the next‐generation matrix method (see Appendix S4) (Diekmann and Heesterbeek 2000) for calculating daily, temperature‐dependent *R*_0_ to arrive at:

(4) $$\;R_{0}\left(t\right)=\sqrt{\frac{\beta_{\mathrm{hv}}(T)\cdot \beta_{\mathrm{vh}}(T)\cdot N_{V}\cdot N_{H}}{{\left(N_{H}+N_{C}\right)}^{2}}\cdot \frac{1}{\left(\mu_{h}+v_{h}+\gamma \right)}\cdot \frac{q(T)}{\mu_{v}(T)\left(\mu_{v}(T)+q(T)\right)}}$$

Here, (*T*) indicates that the parameter is a function of daily temperature. We used the maximum value of daily *R*_0_(*t*) over the annual cycle (peak *R*_0_(*t*)) and the number of days transmission is possible (*R*_0_(*t*) > 1) to evaluate how transmission potential changes under warming.

#### 2.6.2 Infection Outcomes

On the first day on which sustained transmission is possible (i.e., *R*_0_(*t*) > 1), we introduced one infected vector to the breeding site. We solved our model to track emerging infection dynamics for a single bird breeding season, recording daily infection prevalence in the vector population (*I*_*V*_/*N*_*V*_), host infection prevalence (*I*_*H*_/*N*_*H*_), and the proportion of hosts in the infected or recovered classes ((*I*_*H*_ + *R*_*H*_)/*N*_*H*_). To capture the combined costs of infection and resource mismatch in hosts under each warming scenario, we calculated the proportionate decrease in host abundance after pathogen introduction. This was calculated as the daily host abundance (with disease) divided by the historical, pre‐warming daily host abundance prior to pathogen introduction.

### 2.7 Sensitivity Analyses

To determine how vector and pathogen physiological parameters influence transmission potential, we assessed the sensitivity of daily *R*_0_(*t*) to temperature dependence in vector mortality (*μ*_*v*_), biting rate (*α*), and pathogen development rate (*q*) (following Mordecai et al. 2017; Miazgowicz et al. 2020). Each of these traits was held at a constant value (*x* at the mean breeding season temperature within each climate scenario; 15.1°C, 17.7°C, and 20.5°C for historical, RCP 4.5, and RCP 8.5, respectively) while other parameters varied with temperature. This approach allowed us to evaluate the impact of a single physiological trait on transmission potential.

Since it is unclear how climate warming will influence the magnitude of the bird fecundity cost due to mismatch (*c*) or the peak number of vectors emerging (*ε*_max_), we assessed the sensitivity of our model outcomes to a range of values of *c* (corresponding to a 0% to ~25% reduction in maximum host reproduction rate, within the bounds predicted by (Youngflesh et al. 2023)), and *ε*_max_, which we varied to increase or decrease maximum emergence by up to 20% under severe warming. We assumed that the change in *ε*_max_ would scale with the difference in peak temperature between warming and baseline scenarios (see Appendix S6 for more details).

To assess the role of vector‐host overlap in shaping infection outcomes independent of costs to host reproduction (*c*) or disease‐induced mortality (*ν*_*h*_), we also ran the model with either *c* or *ν*_*h*_ set to zero. Finally, when birds arrive earlier under warming in the main model, they breed for a longer period of time (perhaps reflecting opportunities for renesting or multiple broods (Morrison et al. 2019)). To understand the implications of this assumption, we evaluated model outcomes when the host breeding window was kept constant under warming (i.e., breeding stopped before *h*_end_ in scenarios with earlier host arrival) (see Appendix S9 for more details).

Finally, to assess how transmission potential would differ when vector bites are diluted by non‐competent hosts in the community, we ran the model with *N*_C_ = 200 and *N*_C_ = 800 (25% and 100% of the starting focal host population, respectively; see Appendix S10 for more details).

## 3 Results

### 3.1 Effects of Phenology and Warming Scenarios on Disease‐Free Dynamics

**FIGURE 2:**
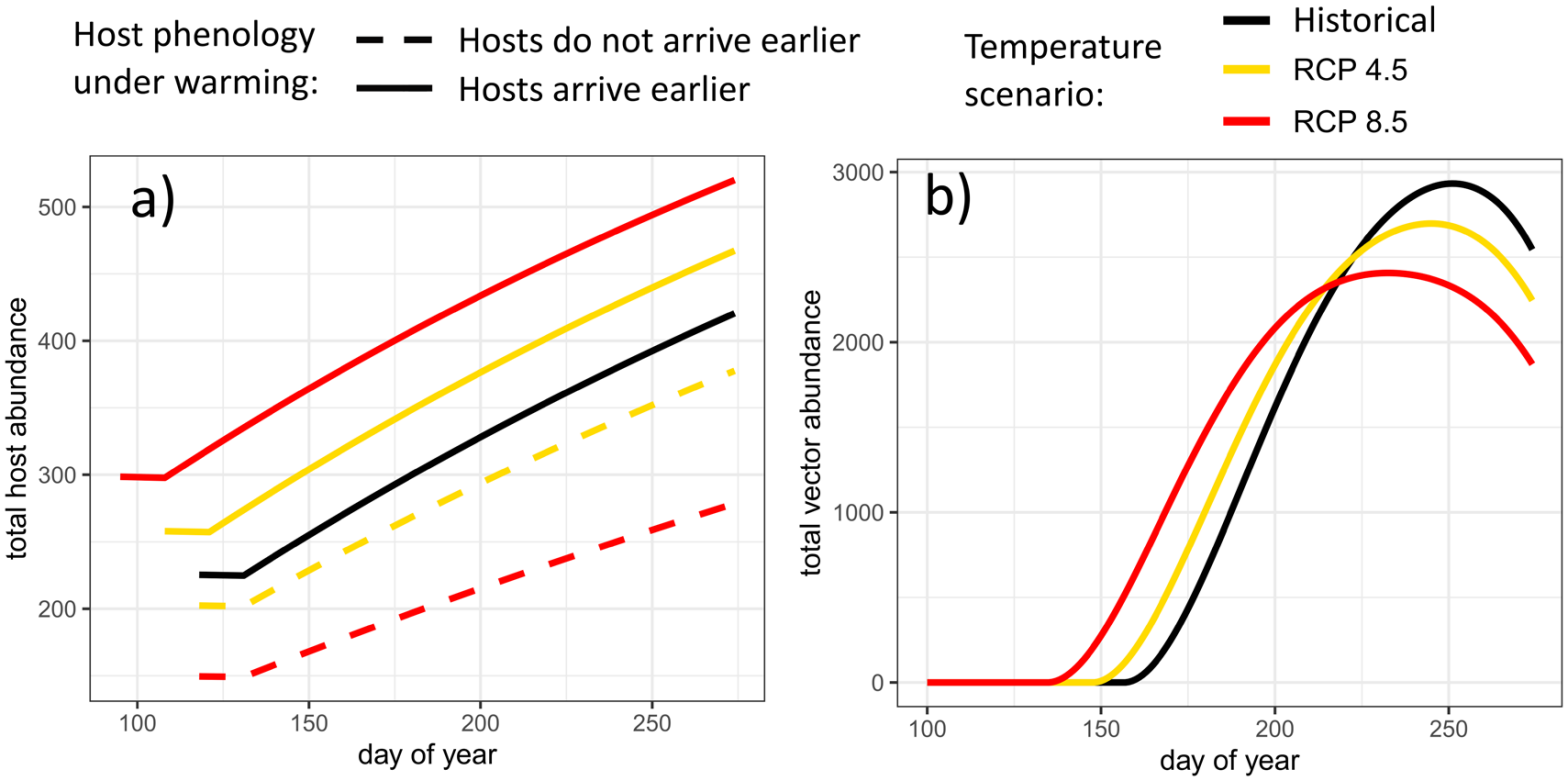
Disease‐free breeding season abundances for (a) hosts and (b) vectors. Colors represent the temperature scenarios: Black = historical baseline, yellow = RCP 4.5 (+2.6°C), red = RCP 8.5 (+5.4°C). For hosts (a), line types represent phenology scenarios: Dashed = hosts do not advance arrival timing with warming, solid = hosts arrive earlier under warming.

In the baseline, historical temperatures scenario, modeled birds arrived in late April, establishing nest territories and incubating eggs before new susceptible birds began hatching in mid‐May. After 30 years and a nonbreeding survival probability of 0.51 (DeSante et al. 2015), the host population settled into a repeating annual cycle with 225 birds returning to colonize the breeding site and yielding 420 birds at the end of the season (Figure 2a).

#### 3.1.1 If Host Arrival Phenology Does Not Change With Warming

Under both moderate and extreme warming scenarios, if hosts did not advance their arrival date, they incurred a reproductive cost that reduced host abundance, with increasing costs tracking the severity of warming. After entering repeating annual population cycles, the peak host population size was 10.2% lower in the moderate warming scenario, and 33.7% lower in the severe warming scenario, relative to the historical peak (Figure 2a, dashed lines).

#### 3.1.2 If Hosts Arrive Earlier Under Warming

When hosts advanced their phenology to track warming temperatures, we assumed earlier arrival led to a longer breeding season, which resulted in higher host abundances. In the moderate warming scenario, birds arrived 10 days earlier and showed an 11.1% increase in abundance relative to the historical scenario, while in the severe warming scenario, birds arrived 23 days earlier and showed a 23.7% increase in host abundance (Figure 2a, solid lines).

#### 3.1.3 Vector Abundance Across Warming Scenarios

Vectors advanced their emergence phenology by 9 days under moderate warming and 22 days under severe warming relative to the baseline scenario, leading to higher vector abundance earlier in the year (Figure 2b). Later in the season, however, the vector population decreased with warming due to shorter adult lifespans at higher temperatures (Figure 2b; Figures S2–S5). Vector abundance peaked 8% and 18% lower in the moderate and severe warming scenarios, respectively.

### 3.2 Effects of Phenology and Warming Scenarios on Transmission Potential

**FIGURE 3:**
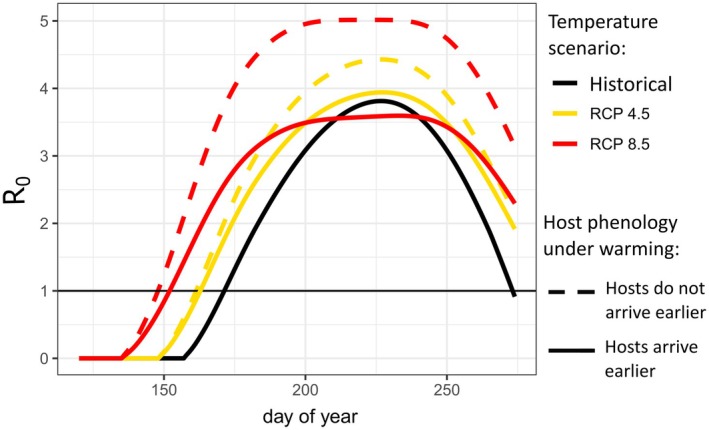
Transmission potential prior to pathogen introduction for each of the five scenarios. Colors represent the temperature scenarios: Black = historical baseline, yellow = RCP 4.5, red = RCP 8.5. Line types represent phenology scenarios: Dashed = hosts do not advance arrival timing with warming, solid = hosts arrive earlier under warming. At values above the horizontal line (*R*_0_(*t*) = 1), sustained transmission is possible.

In most warming scenarios, higher temperatures increased peak R_0_(*t*) relative to the baseline scenario while also increasing the number of days for which transmission was possible (i.e., R_0_(*t*) > 1) (Table 2, Figure 3). Notably, peak R_0_(*t*) was slightly (6%) lower than the baseline scenario when birds arrived earlier under severe warming (Table 2, Figure 3, solid red line). When birds did not advance their arrival phenology, host abundance was substantially lower than in the baseline scenario, leading to a higher vector‐to‐host ratio throughout the season. Thus, individual hosts received more bites, and daily transmission potential was higher relative to when bird arrival tracked warming (Figure 3; dashed vs. solid lines).

**TABLE 2:** Results summary containing scenario‐specific transmission potential metrics and infection outcomes for both vectors and hosts.

Scenario	Peak *R*_0_(*t*)	Number of days *R*_0_(*t*) > 1	Peak vector infection prevalence (*I*_*V*_/*N*_*V*_)	Host infection prevalence (*I*_*H*_/*N*_*H*_) (Peak, Final)	Final cumulative host exposure ((*I*_*H*_ + *R*_*H*_)/*N*_*H*_)	Proportionate reduction in pre‐warming, disease‐free host abundance (post‐pathogen introduction)
(1) Historical temperatures and baseline host and vector phenology	3.813	102	0.014	0.021, 0.018	0.093	0.04
(2) Moderate warming, hosts arrive on historical date, advanced vector emergence	4.430	113	0.188	0.181, 0.115	0.812	0.34
(3) Severe warming, historical host arrival, advanced vector emergence	5.015	127	0.312	0.301, 0.029	0.970	0.56
(4) Moderate warming, earlier host arrival, advanced vector emergence	3.941	112	0.097	0.101, 0.095	0.432	0.06
(5) Severe warming, earlier host arrival, advanced vector emergence	3.595	123	0.255	0.174, 0.115	0.822	0.10

Under more severe warming, we observed a dampened mid‐season peak in *R*_0_(*t*) (Figure 3). Sensitivity analyses show that shorter vector lifespans at high summer temperatures drove the flattening of the peak (Figure S4). Lower vector lifespans reduce both the probability of vectors surviving long enough to transmit and the vector‐to‐host ratio. When hosts tracked extreme warming by advancing their arrival date, this decreased the vector‐to‐host ratio even further, meaning that peak transmission potential was lower under severe warming than under moderate warming (Figure 3, solid red and yellow lines).

### 3.3 Consequences of Pathogen Emergence for Hosts

#### 3.3.1 Host Abundance and Infection

**FIGURE 4:**
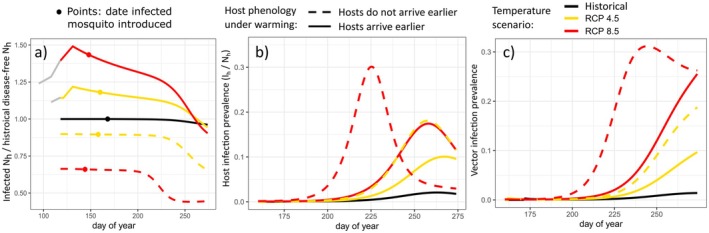
Impacts of pathogen introduction across scenarios. (a) Proportionate decline in host abundance after virus introduction relative to disease‐free historical scenario, (b) host infection prevalence, the proportion of hosts in the infected class, and (c) vector prevalence, the proportion of vectors in the infected class. Colors represent the temperature scenarios: Black = historical baseline, yellow = RCP 4.5, red = RCP 8.5. Line types represent phenology scenarios: Dashed = hosts do not advance arrival timing with warming, solid = hosts arrive earlier under warming. In panel (a), points denote the day the pathogen was introduced for each scenario (first day *R*_0_(*t*) > 1).

To track the initial impacts of a pathogen invading a naïve host population, we introduced a single infected vector on the first day that transmission was possible (i.e., R_0_(*t*) > 1), and solved the model over a single breeding season. Host abundance followed a similar seasonal pattern to the disease‐free dynamics, increasing monotonically to peak abundance at the onset of fall migratory departure. In the historical scenario, 9.3% of hosts were in the infected or recovered classes by the end of the breeding season, which is within the range of American robin seroprevalence values reported by a recent meta‐analysis (1%–17%) (Jahn et al. 2025). More hosts became infected with the pathogen with increasing warming, and host infection prevalence was also higher in scenarios where hosts did not advance arrival phenology, peaking at 30% in the more severe warming scenario (Table 2, Figure 4b).

To assess the combined costs of resource mismatch and infection under warming on host population dynamics, we calculated the ratio of host abundance after pathogen introduction to pre‐warming, disease‐free host abundance (Table 2, Figure 4a). For all scenarios, following pathogen introduction, host relative abundance declined. When hosts advanced their arrival dates to track warming, they gained an early season benefit from advanced reproduction, but earlier onset of pathogen transmission led to declines that yielded end‐of‐season abundances close to that of the historical temperature scenario (Figure 4a). When birds did not advance their arrival timing, the costs of phenological mismatch and infection compounded. End‐of‐season population sizes were 34% and 56% lower than the historic disease‐free abundance for moderate and severe warming scenarios, respectively (Table 2, Figure 4a).

### 3.4 Consequences of Pathogen Introduction for Vectors

In our historical scenario, vector infection prevalence peaked at 0.014, which is within the range of *Culex* mosquito WNV prevalence reported by empirical studies (0.01–0.02) (Chen et al. 2013; Dunphy et al. 2019). Vector prevalence increased under moderate and severe warming. Scenarios where host phenology did not advance generally led to higher vector prevalence (Figure 4c). In all but one scenario, vector prevalence increased until the end of the bird breeding season, but if birds did not advance their arrival under severe warming, vector prevalence peaked much earlier (28 days before the birds migrated). This is likely due to susceptible host depletion, as nearly all hosts were either infected or recovered and immune by this day (Figure 4c).

### 3.5 Sensitivity of Model Outcomes to Model Assumptions

**FIGURE 5:**
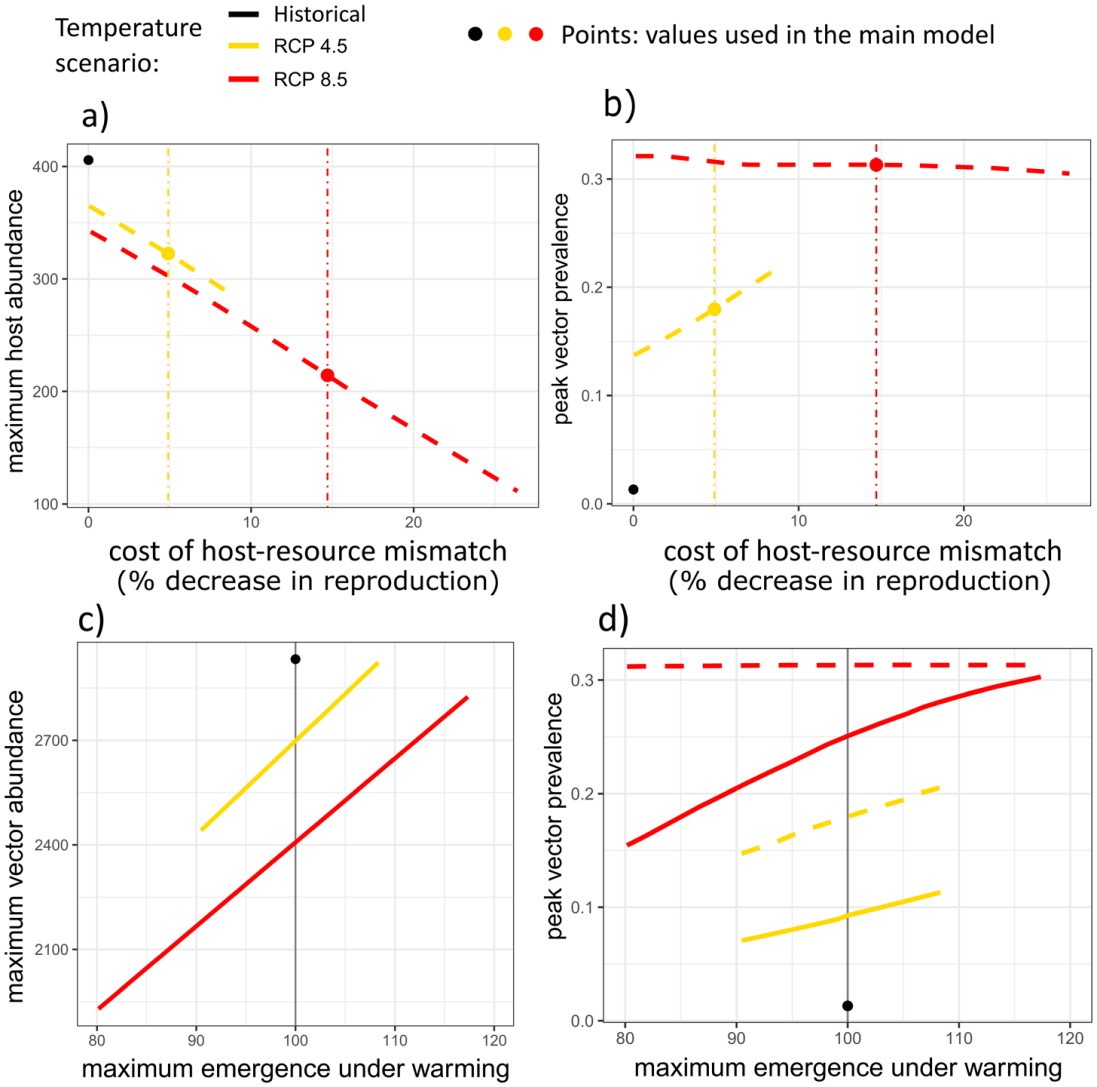
Sensitivity of (a) maximum host abundance and (b) peak vector prevalence to the reproductive cost of host‐resource mismatch. Reproductive costs only apply to scenarios in which birds *do not* advance arrival. Moderate (RCP 4.5, yellow dashed line) and severe (RCP 8.5, red dashed line) warming scenarios are shown. Points and vertical dotted lines represent the values from the main model (black = historical baseline, yellow = RCP 4.5, red = RCP 8.5). Sensitivity of (c) maximum vector abundance and (d) peak vector prevalence to changes in maximum vector emergence rate (*ε*_max_) under moderate (RCP 4.5, yellow lines) and severe (RCP 8.5, red lines) warming scenarios. Line types represent phenology scenarios: Dashed = hosts do not advance arrival timing, solid = hosts arrive earlier under warming. Black points represent the historical baseline scenario, and the gray vertical line at *ε*_max_ = 100 intersects scenario lines at the main model outcomes.

To assess how the cost of host‐resource mismatch impacts outcomes, we varied the cost parameter, *c*. In scenarios where hosts did not advance their arrival phenology, we explored a 0%–25% decrease in the maximum host reproduction rate. As the cost of mismatch increased, maximum host abundance declined (Figure 5a). While peak infection prevalence in vectors increased with the cost of mismatch under moderate warming (Figure 5b), peak vector prevalence declined weakly with increasing mismatch costs under severe warming, possibly because of susceptible host depletion (Figure 5b). Additional exploration of infection dynamics with *c* set to zero indicated that changes to vector‐host overlap under warming matter independent of a fecundity cost (Figure S6). When *c* was set to zero, higher resulting host abundance led to slower infection spread. Notably, in severe warming scenarios without earlier host arrival, vector prevalence did not peak earlier if we removed the fecundity cost (Figure S6).

Peak vector abundance increased approximately linearly with the peak vector emergence rate, *ε*_max_, (Figure 5c), as did peak vector prevalence under moderate warming (Figure 5d). However, when hosts did not advance their arrival date under severe warming, peak vector prevalence did not vary with *ε*_max_, again reflecting susceptible host depletion as vector prevalence reaches about 30%.

When disease‐induced mortality was set to zero, we saw increased transmission despite higher numbers of recovered hosts, likely because infected individuals survived for the entire infectious period (Figure S6). When we held the length of the breeding season constant under warming, qualitative patterns of seasonal prevalence remained the same (Figure S7). When we increased the number of non‐competent hosts (*N*_*c*_), differences between scenarios were dampened, with no differences between warming scenarios when *N*_*c*_ = 800 (Figure S8).

## 4 Discussion

The response of species interactions to climate warming will be shaped by both physiological and phenological responses to temperature shifts, but these effects are rarely studied together. We developed a model to explore how warming influences the dynamics of a hypothetical emerging vector‐borne disease in a migratory host. We found that both shifts in host breeding phenology and warming severity altered the overall amount of infection and its seasonal timing. Temperature‐driven changes in vector physiology and earlier vector emergence phenology generally led to increased transmission and prevalence under warming, but phenological mismatch between hosts and their resources resulted in additional negative fitness impacts on the host population. Notably, severe warming and host phenological mismatch advanced peak infection prevalence in vectors, with late season declines in transmission resulting from depletion of susceptible hosts and elevated vector mortality in the hottest part of the season. While the magnitude of our observed effects is likely determined by our parameterization and model assumptions, this work highlights that accounting for both changes in phenology and physiology can substantially alter infection dynamics under warming, and provides a novel prediction that the inability of migratory hosts to keep up with advancing breeding site phenology can lead to a “double whammy” of phenological resource mismatch and increased infection.

In our model, increased warming and the inability of birds to advance their arrival date led to more infection in hosts. This can largely be explained by an increased vector‐to‐host ratio early in the breeding season, where lower host reproduction due to phenological mismatch was compounded by earlier vector emergence and increased vector competence for infection. Our findings differ from another model exploring host and vector phenology, in which host prevalence decreased with increasing host phenological mismatch (Hall et al. 2016). In that model, however, both vector emergence and host reproduction occurred in a single pulse, so when birds did not arrive earlier, temporal overlap between vectors and susceptible hosts decreased. In contrast, if hosts did not arrive earlier under warming in our model, advancing vector phenology increased their temporal overlap with hosts and led to more bites per host due to low host density and higher, temperature‐dependent biting rates. Similarly, a field‐informed model of blood parasite infections in white‐crowned sparrows found that advancing blackfly vector emergence increased prevalence in birds (Murdock et al. 2013). Together, these suggest that synchronicity of host breeding and vector emergence is a crucial determinant of host–parasite responses to warming.

### 4.1 Implications for Migratory Hosts

Our findings indicate that emerging infectious diseases can present an important challenge to migratory birds in future climate regimes. Emerging pathogens can cause declines in wildlife populations through disease‐induced mortality, especially after a recent introduction (LaDeau et al. 2007; Foppa et al. 2011; George et al. 2015). We found that warming‐induced phenological mismatches between hosts and resources increased the prevalence of a virulent pathogen, exacerbating negative impacts on host abundance. In American robins, temperature and snow cover along the migration route are predictors of spring arrival phenology (Travers et al. 2015; Oliver et al. 2020), suggesting some plasticity to track earlier warming. Although many temperate‐breeding birds have been arriving earlier or reducing the time between arriving and first nesting (Both et al. 2004; Miller‐Rushing et al. 2008; Travers et al. 2015), some long‐distance migrants are still not able to keep up with the pace of climate warming (Visser and Both 2005; Both et al. 2006; Saino et al. 2011). Species that use cues such as photoperiod to time their departure from non‐breeding sites, or that make few to zero stopovers on their pre‐breeding migration, may be especially vulnerable to mismatch‐related declines (Taylor et al. 2016) and additional disease‐related mortality if vectors exhibit strong biting preferences. Additionally, birds that are dietary specialists might be more likely to experience mismatch‐induced population declines, as they would be less able to shift to an alternate food resource (Johansson et al. 2015). Birds breeding at high latitudes or those capable of adjusting migratory phenology might also experience unprecedented vector pressure due to vector range expansions or extended windows of biting activity (Møller 2010; Brown and Hall 2018). Finally, if an extended bird breeding window enables breeding pairs to produce more broods per season, parasite transmission could rise through an increase in highly susceptible young (De Lope and Møller 1993; Hall 2021).

Some wildlife species demonstrate a decrease in migratory behavior in response to climate change (Visser and Both 2005; Wilcove and Wikelski 2008; Pulido and Berthold 2010). Some species, like the American robin, exhibit partial migration, with some individuals completing long‐distance migrations and others remaining year‐round residents (Brown and Miller 2016; Jahn et al. 2019). Migratory propensity can impact infectious diseases (Satterfield et al. 2015; Bergsman et al. 2016; Brown and Hall 2018; Peacock et al. 2022), and infection can, but does not always, feed back to influence migratory behavior (Nendick et al. 2011; Narayanan et al. 2020; Hall et al. 2022; Teitelbaum et al. 2023). In the model developed by Brown and Hall (2018), increased temporal overlap between vectors and resident birds under warming also increased transmission, eroding the benefits of migratory escape for migrants and jeopardizing the persistence of migration. Further, if infection also reduces migration probability, reinforcing feedback between reduced migration and increased parasitism in residents could exacerbate the loss of migration (Hall et al. 2022). Alternatively, if resident breeding and vector emergence peak before migrants arrive, this could facilitate the coexistence of highly parasitized residents and low‐prevalence migrants (Emmenegger et al. 2018).

### 4.2 Future Applications

The modeling framework presented here could be adapted to explore other questions relating to vector‐borne disease risk under climate change. In addition to the vector community, host community composition, variation in host phenology, and differences in host competence for infection could be used to explore how wildlife diversity and specific species contribute to pathogen transmission in the enzootic cycle (Bergsman et al. 2016). Age structure in host populations could also be incorporated, especially if hatch‐year birds are more susceptible or have higher migration mortality rates (Hamer et al. 2008; Murdock et al. 2013). Additionally, while we only explored phenological responses of endothermic hosts in this model and potential costs to reproduction, in some cases, it might also be appropriate to consider host immunological responses to climate change (Hall 2019). For example, Owen and colleagues found that if infected American robins were food‐stressed, which could occur with phenological mismatch, the probability of transmission to a susceptible mosquito increased (Owen et al. 2021).

Mathematical models could also consider other climate scenarios and additional variables that predict host, vector, or parasite traits. Some studies explore scenarios in which temperatures increase more rapidly in winter months (e.g., (Gehman et al. 2018)), while others could consider scenarios with more variable temperatures. If immature vector stages were modeled explicitly, changes in precipitation or humidity could be used to predict how climate change would impact these stages. Furthermore, we focused on the thermal responses of biting adults, but explicitly modeling temperature effects on immature development and survival could also impact patterns of adult abundance and vector competence under warming (Ewing et al. 2016; Evans et al. 2018, 2019). We only explored phenological responses in spring vector emergence, but climate warming could also extend vector biting activity later into the fall. The fraction of diapausing mosquitoes, those that emerge in a state of delayed reproduction for overwintering, can depend on temperature (as modeled in (Rubel et al. 2008)), but mosquito vectors also rely on photoperiod to initiate wintering physiology and behavior (Field et al. 2022). Field‐ or experiment‐informed data on vector biting activity and diapause should be used to model late‐season vector phenology and physiology under climate change. A recent synthesis paper described important considerations and modeling approaches for forecasting mosquito activity patterns under future climate conditions (McDevitt‐Galles et al. 2024). They noted that many models do not address the roles of adaptation and phenotypic plasticity in shaping future responses to climate change (Couper et al. 2021; Zettlemoyer and Peterson 2021; Abbasi 2025). For mosquito vectors specifically, adaptation to elevated temperatures is certainly possible. Strong trait‐temperature relationships will yield selective pressures under warming, and population‐level differences in thermal performance suggest that the genetic variation needed for adaptation exists (Couper et al. 2021) (Table S2).

Because our questions were focused on pathogen emergence in a naive host, we only followed infection dynamics over a single breeding season. Future modeling efforts could assess the longer‐term effects of phenological and physiological responses on transmission throughout the annual cycle. For instance, interannual disease dynamics will be shaped by the proportion of birds that recover with immunity and the timing of breeding, as both factors impact the number of susceptible hosts. It is not common for seroprevalence to reach 100% in the field (Jahn et al. 2025), so our finding that nearly all hosts are exposed to the pathogen under severe warming likely reflects some of the model assumptions, including no prior host immunity, the effects of severe warming, and preferential biting of a single host species. Multi‐site models could also be developed to assess how climate impacts disease across the host's range, including at stopover and wintering sites. For WNV specifically, the relative importance of mechanisms leading to endemic infection is not well understood (Reisen 2013). Recrudescing chronic infections in hosts can initiate transmission cycles (Becker et al. 2020), and it is possible for viruses to survive in overwintering mosquitoes (due to vertical transmission or survival of blood‐fed females) (Cruz‐Pacheco et al. 2005). Models could explore how each of these potential mechanisms would influence transmission under climate warming. Finally, if this modeling framework were being used to predict infection outcomes in a specific region or for a specific disease system, the degree day calculation should be tailored to the species and should not assume linear relationships between degree days and the traits in question. It would also be beneficial to use more temporally fine‐scale temperature measurements, as invertebrate traits are responding to continuous temperatures, not just daily mean temperatures.

## 5 Conclusions

Incorporating endothermic host phenological responses to climate change impacted vector‐borne disease dynamics, with large effects on transmission and the host population. This finding is significant because most models of vector‐borne disease to date have focused on the thermal responses of physiological traits. Responses of wildlife pathogens to climate change can have implications for ecosystem services, conservation, and public health, so it is important to develop predictive frameworks that can incorporate multiple species' responses to novel climates. Here, we describe the importance for host‐pathogen outcomes, but this approach could be extended to other species interactions involving temperature‐sensitive or highly mobile species like plant‐pollinator (Fagan et al. 2014; Iler et al. 2021), predator–prey (Hall et al. 2006; Iler et al. 2021), consumer‐resource (Iler et al. 2021; Kharouba and Yang 2021), and interspecific competition (Iler et al. 2021; Pardikes et al. 2022).

## Author Contributions

**Isabella G. Ragonese:** conceptualization (equal), formal analysis (lead), funding acquisition (equal), methodology (equal), software (equal), visualization (lead), writing – original draft (lead), writing – review and editing (equal). **Sonia Altizer:** conceptualization (equal), methodology (supporting), supervision (supporting), writing – review and editing (supporting). **Courtney C. Murdock:** conceptualization (supporting), methodology (equal), supervision (supporting), writing – review and editing (equal). **Richard J. Hall:** conceptualization (equal), formal analysis (equal), methodology (lead), project administration (equal), supervision (lead), visualization (equal), writing – original draft (supporting), writing – review and editing (equal).

## Funding

This work was supported by the National Science Foundation [Graduate Research Fellowship #1842396 and Research Traineeship DGE‐1545433 to I.G.R; DEB‐1911925 to R.J.H.]. The funders had no role in study design, data collection and analysis, decision to publish, or preparation of the manuscript.

## Conflicts of Interest

The authors declare no conflicts of interest.

## Supporting information


**Appendix S1:** Supporting Information.

## Data Availability

The model code and outputs are archived in the Dryad Digital Repository: https://doi.org/10.5061/dryad.cnp5hqcdn. Code is also available in a GitHub repository: https://github.com/IRagonese/VBD_phenology_physiology_2024.
