# Supplementary material for: Modeling Phenological and Physiological Responses to Climate Warming in a Hypothetical Migratory Songbird–Mosquito System
Source: Ecol Evol. 2025 Dec 11;15(12):e72648. doi: 10.1002/ece3.72648 (PMC12696666; doi:10.1002/ece3.72648)
Supplement: Supplementary file 1 — Appendix S1: Supporting Information. [file ECE3-15-e72648-s001.docx]

**Appendix**

To accompany: “Modeling Phenological and Physiological Responses to Climate Warming in a Hypothetical Migratory Songbird–Mosquito System” in *Ecology and Evolution*

Authors: Isabella G. Ragonese, Sonia Altizer, Courtney C. Murdock, Richard J. Hall

1. ***Model parameterization - hosts***

We based the full annual cycle dynamics of our migratory host species on the American Robin (*Turdus migratorius*), an abundant migratory species that has been implicated as an important host for WNV (Kilpatrick et al., 2006). At the northern edge of their breeding range, robin populations are fully migratory (Vanderhoff et al., 2016). Although our model only considers breeding season dynamics in the year following WNV introduction, we built a full annual cycle model to determine the number of adult birds returning to the breeding site under each of our climate scenarios.

*Season-specific host survival:* Robins have an approximate annual survival, *σ_a_* ≈ 0.51 (DeSante et al., 2015; Brown and Miller, 2016; Vanderhoff et al., 2016; Brown and Hall, 2018), which can be decomposed into the probability of surviving the breeding (b) and nonbreeding (nb) seasons, i.e. *σ_a_* = *σ_b_ σ_nb_*. Prior to climate warming and WNV introduction, we assumed a high probability of survival of birds on the breeding ground, *σ_b_* = 0.97, which results in a nonbreeding survival of *σ_nb_* = 0.52. We assume birds experience mortality at a constant per capita rate in the breeding and nonbreeding seasons, so that the number of individuals surviving from the start of season j (= b or nb) is described by

Eq. (S1) $\frac{dN_{H}}{dt}=-\mu_{h,j}N_{H}$

We calculated the annual per capita mortality rates for the breeding (*μ_h,b_*) and nonbreeding (*μ_h_nb_*) seasons, by rearranging the expression *s_j_* = $e^{-\mu_{h,j}T_{j}}$, where *T_j_* is the time spent in season j. (L. M. Brown & Hall, 2018).

*Host fecundity:* We assumed that hosts had a per capita offspring production rate that decreases linearly with host density (*b_0_*-*b_1_*N). We assumed that paired hosts have a maximum of approximately three broods per year with three individuals fledged per nest (Vanderhoff et al., 2016), for a maximum per capita fecundity, *F_max_*, ≈ 4.5, and estimated *b_0_* from the expression for the maximum offspring production over the breeding season in the absence of density dependence, *F_max_* = exp(*b*_0_T*_b_*). We calculated the scaling effect of density on the birth rate (*b_1_*) by assuming that the birth rate becomes 0 when there are ~1000 individuals in the breeding population (Hall et al., 2016) yielding *b_1_ = b_0_/*1000. We assumed that birds take approximately two weeks after arrival on the breeding site to set up breeding territories and incubate eggs (Vanderhoff et al., 2016), and therefore estimated the number of breeding birds each timestep by reducing the number of returning hosts (*N_start_*) by the per capita breeding mortality rate for the number of days since host arrival (*h_start_*):

Eq. (S2) *N_breed_* = *N_start_* ⋅ $e^{(-\mu_{h}\cdot\left( t-h_{start} \right))}$

*Costs of WNV infection to hosts:* Compared to other North American birds like corvids, American Robins are less likely to succumb to infection with WNV (Komar et al., 2003), but as our bird population is naïve and because WNV has been implicated in American Robin declines (LaDeau et al., 2007), we assumed that some robins die due to infection. Given an infectious period of 5.5 days (Rubel et al., 2008; Owen et al., 2021), daily removal rate from the infected class was set at 1/5.5 = 0.182. Following prior models of WNV and related viruses (Rubel et al., 2008), we assumed that about 30% of birds infected with WNV die from infection, so disease-induced mortality, *ν_h_*, equals (0.3)⋅(0.182) = 0.055. This value falls in the range explored in (Bergsman et al., 2016), as well. The recovery rate of birds, γ, is then approximately (1 - 0.3)⋅(0.182) ≈ 0.1 (Rubel et al., 2008; Laperriere et al., 2011; Bergsman et al., 2016).

1. ***Model parameterization – vector and pathogen***

For temperature-dependent parameters (Table S1), we used functions presented in (Shocket et al., 2020) describing WNV infections in *Culex tarsalis*.

**Table S1.** Parameters describing the thermal performance of WNV and *Culex tarsalis* traits. Functional forms are Briere: Q⋅temp ⋅ (temp-T_min_) ⋅ (sqrt(T_max_-temp)) and Linear: (-m⋅temp)+z.

| **Trait** | **Function** | **Q** | **T_min_** | **T_max_** | **T_opt_** | **Source** |
| --- | --- | --- | --- | --- | --- | --- |
| biting rate | Briere | 1.67x10^-4^ | 2.3 | 32 | 25.9 | (Reisen et al., 1992; Shocket et al., 2020) |
| pathogen development rate | Briere | 6.57x10^-5^ | 11.2 | 44.7 | 37 | (Reisen et al., 2006; Shocket et al., 2020) |
| lifespan | Linear | m=1.69 | z=69.6 | 41.3 | - | (Reisen, 1995; Shocket et al., 2020) |

1. ***Model parameterization – hosts and vector phenology under climate warming***

Under baseline conditions and moderate or severe climate warming scenarios, we used Growing Degree Days to calculate the optimal emergence dates for vectors. We calculated degree day using the Growing Degree Days (‘gdd’) function in the *pollen* package in R (Nowosad, 2021). We set tbase=0, type=“C” and tbase_max=40 (as the thermal maxima for vector and parasite traits are close to 40°C). We applied this ‘gdd’ function to calculate the cumulative degree days as a function of temperature data for our historical breeding site temperatures over an annual cycle, and projected temperatures under the RCP4.5 and RCP8.5 warming scenarios (Figure S1).

We assumed that the pathogen is transmitted by adult *Culex sp.* mosquitoes with a seasonal emergence window during the favorable season at host breeding sites (i.e., emergence beginning when cumulative degree days above 0°C reach 312) (Bolling et al., 2007), with peak emergence occurring in the middle of the season. Following (Hall et al., 2016) and (Brown and Hall, 2018), we assumed a peak vector emergence rate of approximately 100/day. In RCP 4.5 and RCP 8.5 warming scenarios, vector emergence advances, as 312 degree days are reached on an earlier date.

Based on empirical data tracking robin spring arrival dates, we assume the historical bird arrival date tracks the 2.8°C isotherm (Bent, 1949). If birds continue to track this isotherm under warming, their arrival dates under RCP4.5 and 8.5 are the respective first days of the year that mean temperatures pass this threshold. Since robins rely on invertebrate prey to feed their offspring, and invertebrate availability is associated with cumulative degree days (Shaftel et al., 2021) we assume that birds experience a fecundity cost that scales with the difference in cumulative degree days from their historical host arrival date (dd_0_), and the degree days on their arrival under warming (dd_j_; j= [4.5, 8.5]) (Figure S1).


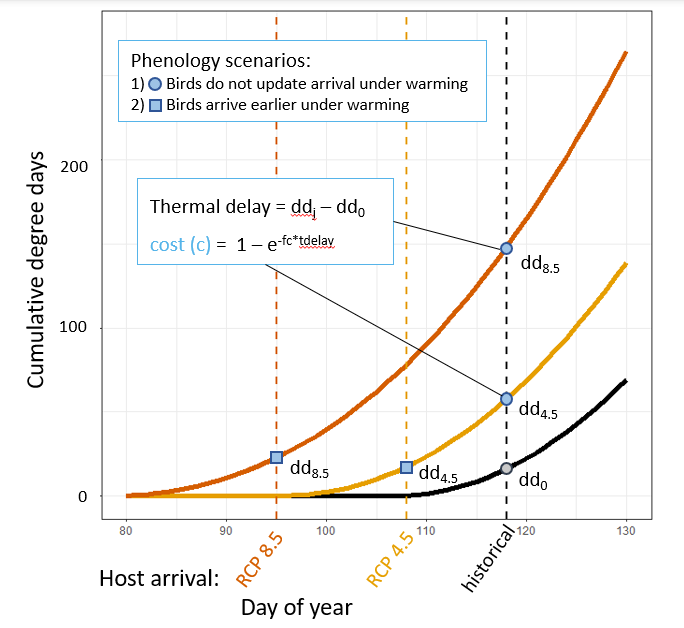


**Figure S1.** Cumulative degree days above 0°C across days of the year for each temperature scenario: historical temperatures (black), moderate warming (RCP 4.5, yellow), and severe warming (RCP 8.5, orange). The black dashed vertical line indicates the historical bird arrival date, which is the same for birds under warming if they do not advance phenology (black), and bird arrival dates under moderate (yellow) and severe (orange) warming if they advance phenology to track the 2.8$^{\circ}$C isotherm. Points indicate the degree days on arrival for each of the 5 scenarios, with circles representing no advanced bird arrival and squares representing earlier bird arrival.

1. ***Model analysis: calculating Temperature-dependent relative R_0_***

We calculated the basic reproductive number (R_0_) from the dominant eigenvalue of the next-generation matrix (as in (Diekmann and Heesterbeek, 2000; Wonham et al., 2006; Rubel et al., 2008)). The disease-free equilibrium for the avian host population is defined as (S_H_, I_H_, R_H_) = (N_H_*,0,0) and the mosquito equilibrium is defined as (S_V_, E_V_, I_V_) = (N_V_*,0,0), with N_H_* and N_V_* as the numbers of birds and mosquitoes at disease-free equilibrium.

We used the equations containing infection terms (Main text Eqs. (1b), (1e), and (1f)) to identify the rates of gain and loss of infection:

Eq. 1b) $\frac{{dI}_{H}}{dt}=\frac{\beta_{vh}S_{H}I_{V}}{N_{H}+N_{C}}-{(\mu}_{h}+\nu_{h}+\gamma)I_{H}$

Eq. 1e) $\frac{{dE}_{V}}{dt}=\frac{\beta_{hv}S_{V}I_{H}}{N_{H}+N_{C}}- {(\mu}_{v}+q)E_{V}$

Eq. 1f) $\frac{{dI}_{V}}{dt}=qE_{V}-\mu_{v}I_{V}$

Gains for *I_H_*: $\frac{\beta_{vh}S_{H}I_{V}}{N_{H}+N_{C}}$

Gains for *E_V_*: $\frac{\beta_{hv}S_{V}I_{H}}{N_{H}+N_{C}}$

Gains for *I_V_*: 0 (dependent on *E_V_*)

Losses for *I_H_*: ${(\mu}_{h}+\nu_{h}+\gamma)I_{H}$

Losses for *E_V_*: ${(\mu}_{v}+q)E_{V}$

Losses for *I_V_*: $\mu_{v}I_{V}-qE_{V}$

Matrices F (gains) and V (losses) are derived by taking the partial derivatives of these gain and loss terms with respect to the three state variables, *I_H_*, *E_V_*, and *I_V_*.

$$F=\left[ \frac{\partial}{\partial I_{H}}\left( \frac{\beta_{vh}S_{H}I_{V}}{N_{H}+N_{C}} \right) \frac{\partial}{\partial I_{H}}\left( \frac{\beta_{hv}S_{V}I_{H}}{N_{H}+N_{C}} \right) \frac{\partial}{\partial I_{H}}0 \frac{\partial}{\partial E_{V}}\left( \frac{\beta_{vh}S_{H}I_{V}}{N_{H}+N_{C}} \right) \frac{\partial}{\partial E_{V}}\left( \frac{\beta_{hv}S_{V}I_{H}}{N_{H}+N_{C}} \right) \frac{\partial}{\partial E_{V}}0 \frac{\partial}{\partial I_{V}}\left( \frac{\beta_{vh}S_{H}I_{V}}{N_{H}+N_{C}} \right) \frac{\partial}{\partial I_{V}}\left( \frac{\beta_{hv}S_{V}I_{H}}{N_{H}+N_{C}} \right) \frac{\partial}{\partial I_{V}}0 \right]$$

$$=\left[ \frac{{-\beta}_{vh}S_{H}I_{V}}{{(N_{H}+N_{C})}^{2}} \frac{\beta_{hv}S_{V}}{N_{H}+N_{C}}-\frac{\beta_{hv}S_{V}I_{H}}{{(N_{H}+N_{C})}^{2}} 0 0 0 0 \frac{\beta_{vh}S_{H}}{N_{H}+N_{C}} 0 0 \right]$$

Substituting in the disease-free equilibrium, (*S_H_*, *I_H_*, *R_H_*, *S_V_*, *E_V_*, *I_V_*) = (*N_H_**, 0, 0, *N_V_**, 0, 0), we obtain the corresponding matrices F (gains)

$$F=\left[ 0 \frac{\beta_{hv}{N_{V}}^{*}}{{N_{H}}^{*}+N_{C}} 0 0 0 0 \frac{\beta_{vh}{N_{H}}^{*}}{{N_{H}}^{*}+N_{C}} 0 0 \right]$$

And V (losses),

$$V=\left[ \frac{\partial}{\partial I_{H}}\left( {(\mu}_{h}+\nu_{h}+\gamma)I_{H} \right) \frac{\partial}{\partial I_{H}}\left( {(\mu}_{v}+q)E_{V} \right) \frac{\partial}{\partial I_{H}}{(\mu}_{v}I_{V}-qE_{V}) \frac{\partial}{\partial E_{V}}\left( {(\mu}_{h}+\nu_{h}+\gamma)I_{H} \right) \frac{\partial}{\partial E_{V}}\left( {(\mu}_{v}+q)E_{V} \right) \frac{\partial}{\partial E_{V}}{(\mu}_{v}I_{V}-qE_{V}) \frac{\partial}{\partial I_{V}}\left( {(\mu}_{h}+\nu_{h}+\gamma)I_{H} \right) \frac{\partial}{\partial I_{V}}\left( {(\mu}_{v}+q)E_{V} \right) \frac{\partial}{\partial I_{V}}{(\mu}_{v}I_{V}-qE_{V}) \right]=\left[ \mu_{h}+\nu_{h}+\gamma0 0 0 \mu_{v}+q -q 0 0 \mu_{v} \right]$$

Taking the inverse of the loss matrix V yields:

$$V^{-1}=\left[ \frac{1}{\mu_{h}+\nu_{h}+\gamma} 0 0 0 \frac{1}{\mu_{v}+q} \frac{q}{{(\mu}_{v}+q)\mu_{v}} 0 0 \frac{1}{\mu_{v}} \right]$$

Next, we calculate the matrix M = FV^-1^:

$$M=\left[ 0 \frac{\beta_{hv}N_{V}}{N_{H}+N_{C}}\cdot\frac{1}{{(\mu}_{v}+q)} \frac{\beta_{hv}N_{V}}{N_{H}+N_{C}}\cdot\frac{q}{{(\mu}_{v}+q)\mu_{v}} 0 0 0 \frac{\beta_{vh}N_{H}}{N_{H}+N_{C}}\cdot\frac{1}{{(\mu}_{h}+\nu_{h}+\gamma)} 0 0 \right]$$

To calculate the basic reproductive number, we solve the characteristic equation det(M - 𝜆I) = 0 (where I is the identity matrix) for the eigenvalues, which yields:

$${-\lambda}^{3}+M_{31}\cdot M_{13}\cdot\lambda=0$$

Substituting in for M_31_ and M_13_, and noting that R_0_ is the largest positive eigenvalue, we can rearrange and solve to yield:

$$R_{0}=\sqrt{\frac{\beta_{hv}\beta_{vh}{N_{H}}^{*}{N_{V}}^{*}}{\left( {N_{H}}^{*}+N_{C} \right)^{2}}\cdot\frac{1}{{(\mu}_{h}+\nu_{h}+\gamma)}\cdot\frac{q}{{\mu_{v}(\mu}_{v}+q)}}$$

For each climate scenario, we use this R_0_ expression to calculate a daily effective R_0_, where each temperature-dependent parameter is evaluated at the daily temperature and the *N_H_** and *N_V_** values are the daily disease-free host and vector population sizes, respectively.

1. ***Model assumptions - limitations, implications, and potential model modifications***

**Table S2**. Model assumptions, their limitations and implications. We include information on how we explored model sensitivity to the assumptions or how future models could address additional ecological complexities.

| **Simplifying assumption** | **Limitations & Implications** | **Sensitivity & Future modifications?** |
| --- | --- | --- |
| Birds either keep historical arrival date or respond to track temperature perfectly | We considered these two extremes, but many migratory species might show a partial shift in arrival timing. However, even hosts that partially advance their arrival can exhibit a thermal delay (Saino et al., 2011) | *h_start_* is modifiable and could be adjusted to represent intermediate phenological responses |
| Only the focal host species can acquire and transmit the pathogen, and vectors preferentially bite this host | The presence of other host species could affect seasonal transmission potential and the focal host pathogen exposure. Different patterns would result depending on the other hosts’ competence, density, and vector biting preferences | We partially addressed this assumption in Appendix 10 by running the model with other non-competent host species (N_c_).  Alternate model structures could account for different host species |
| Host individuals have synchronized arrival (*h_start_*) and departure (*h_end_*) from the breeding site | If hosts show staggered arrival or departure, the vector:host ratio during those periods would differ, impacting transmission potential. While the date on which R_0_(*t*) > 1 could shift, there would still be differences between temperature scenarios | Sex (Tøttrup and Thorup, 2008) and age (Stewart et al., 2002) can influence arrival synchrony in passerines, and some species arrive more synchronously. Host arrival or departure could be modeled as a rate within a designated arrival/departure window |
| Vectors always respond phenologically, emerging earlier based on spring degree-day accumulation | Vectors might respond differently if microclimate or photoperiod cues drive phenology. This response impacts the vector:host ratio and seasonal transmission potential | The degree of host and vector overlap matters in past models (Murdock et al., 2013; Hall et al., 2016); Future work could adjust how v*_start_* is modeled based on a focal system |
| Seasonal vector emergence rate, *ε*(*t*)*,* is described by a unimodal, quadratic function with peak emergence *ε_max_* in the middle of the vector activity season | Mechanistic drivers of vector emergence/seasonal abundance (e.g., impacts of host density and breeding site availability on reproduction, effects of photoperiod on diapause) are not modeled explicitly, which limits realism of abundance patterns | For application to a specific vector species, the emergence rate could be revised to produce a specific abundance pattern |
| Vector survival is not blood meal-limited at lower host densities | Vector traits like mortality (and resulting transmission potential) in the early spring could differ if lower host densities interact with thermal performance | Other functional forms of vector mortality, bite rate, and emergence could take host availability into account (Dahlin et al., 2024) |
| Adaptation to climate warming is not modeled | Differences in future thermal performance curves are not considered. Gradual adaptation to warming might include increased vector thermal tolerance or a faster pathogen development rate, dampening the model outcomes presented here | Novel thermal response curves based on theoretical or empirically-informed adaptations could be substituted into the model (Couper et al., 2021) |
| The host population does not include age structure | Age can influence host susceptibility and migration timing. Including highly susceptible hatchlings might amplify observed effects | A future model could include host age structure (e.g., Murdock et al., 2013) but while values for prevalence might change, the patterns presented in the results would remain. |
| Fixed ends of seasonal host breeding and vector activity periods; no temperature-dependence determining these dates | Our phenological scenarios do not capture the full range of phenological responses, but autumn responses of migratory birds are not consistent, and vector diapause is also dependent on photoperiod | We partially addressed this assumption in Appendix 9 by fixing the breeding season duration. *h_end_* and *v_end_* are modifiable and could be adjusted to represent alternative autumn phenology scenarios |
| Hosts experience disease-induced mortality | American robins might exhibit low or no WNV-induced mortality, but infection impacts on survival vary widely and can be especially high for emerging pathogens | We addressed the sensitivity of our model to this assumption (See Appendix 8) |
| Under warming, hosts experience a cost to reproduction if they arrive at a higher degree-day value | Some hosts might show plastic responses that largely buffer against late arrival. | We addressed the sensitivity of our model to this assumption (See Appendix 8) |

1. ***Sensitivity analysis – warming impacts on host fecundity cost (*c) *and maximum vector emergence rate* (ε_max_)**

To assess the impact of our assumptions about the magnitude of the mismatch-induced fecundity cost (*c*) on model outcomes, we varied the parameter *f_c_*, which scales how rapidly reproduction declines with thermal delay:

Eq. (3) $c=1-e^{\left( -f_{c} \cdot t_{delay} \right)}$

where *t_delay_* (= dd_j_-dd_opt_) is the thermal delay. We varied *f_c_* from 0 to 0.0024, which yielded cost values between 0 and 0.26, which are representative of percent reductions in bird fecundity predicted in (Youngflesh et al., 2023).

We also relaxed our assumption that the maximum daily emergence rate of vectors was unaffected by warming, allowing vector emergence to increase or decrease under warming. We modeled this as
Eq. (S3) *ε*_max =_ *ε*_max_base_ + θ • (max(temp_j_) - (max(temp_hist_))

where *ε*_max_base_ is the baseline maximum emergence used in the model, max(temp_j_) and max(temp_hist_) are the respective maximum annual temperatures under warming scenario j(=RCP4.5 or 8.5) and the historic maximum annual temperature, and θ scales the direction and strength of this effect. We varied θ between -4 and 4, leading to range of peak emergence of 80-117 mosquitoes/day (baseline = 100 mosquitoes per day).

We ran the model for all warming and phenology scenarios, first keeping θ constant at 0 and varying *f_c_,* and measured its impacts on maximum host abundance and peak vector prevalence. We then ran the model with *f_c_*=0.0012 (our baseline value) and varied θ, tracking impacts on peak vector abundance and prevalence.

1. ***Sensitivity analysis – impacts of physiological parameters on R_0_***

To understand whether warming effects on the effective reproduction number were driven by temperature effects on a focal parameter, we compared the temperature-dependent R_0_ curves in the presence and absence of temperature effects on that parameter. Each focal trait was held at a constant value (x at the mean breeding season temperature within each climate scenario; 15.1°C, 17.7°C, and 20.5°C for historical, RCP 4.5, and RCP 8.5, respectively) while other parameters varied with temperature, allowing us to evaluate the impact of a single physiological trait on transmission potential.

When biting rate (*α*) is held constant, R_0_ tends to peak lower and later, while late-season transmission potential does not decline as drastically (Figure S2). We still see the depressed peak in transmission potential in the RCP 8.5 scenario. When biting rate is constant and birds arrive earlier under warming, peak R_0_ is lower for the two warming scenarios relative to historical temperatures (Figure S2b).

Without temperature-dependence in pathogen production rate (*q*), peak R_0_ is lower in each respective treatment, but we still observe the depressed peak in R_0_ in the severe RCP 4.5 scenario (Figure S3).

Without temperature-dependence in mosquito lifespan (*ls*) and mortality (*μ_v_*=1/*ls*), peak R0 is higher for the two warming scenarios, and we no longer observe the depressed peak in the RCP 8.5 scenario, indicating that the higher mortality and lower mid-season vector abundance in our model is driving the decrease in transmission potential (Figure S4). Without temperature-dependence in mosquito lifespan (and mortality), mosquito abundance is not suppressed under warming and peaks higher than the historical value (just slightly earlier due to the emergence function) (Figure S5).


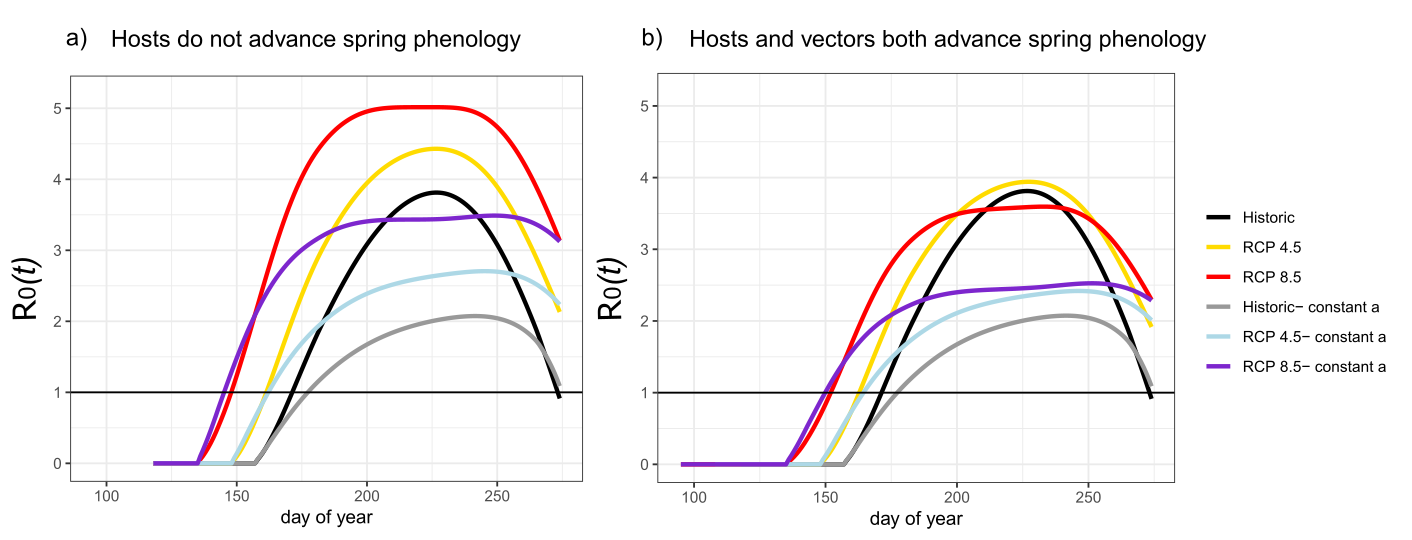
**Figure S2.** Temperature-dependent transmission potential, R_0_, across the breeding season for scenarios in which birds do not arrive earlier under warming (a) and scenarios in which birds advance arrival timing under warming (b). Model scenarios presented in the main text (historical= black; RCP 4.5= yellow; RCP 8.5=red) are depicted relative to scenarios with biting rate (α) held constant (historical= gray; RCP 4.5= blue; RCP 8.5=purple).


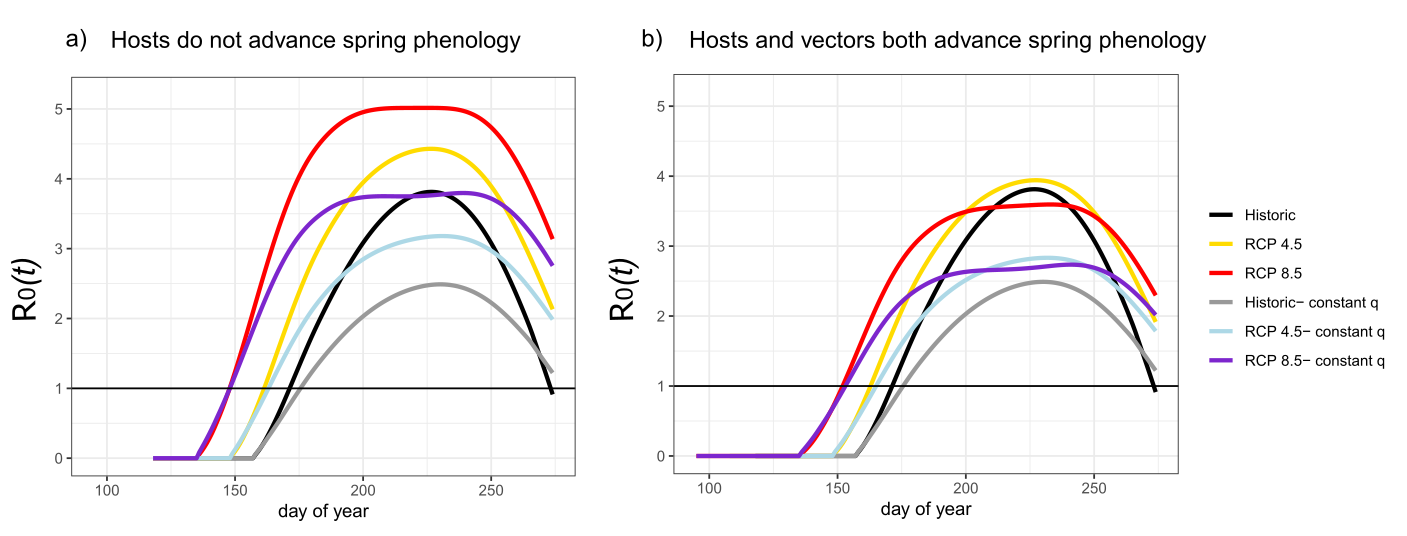
**Figure S3.** Temperature-dependent transmission potential, R_0_, across the breeding season for scenarios in which birds do not arrive earlier under warming (a) and scenarios in which birds advance arrival timing under warming (b). Model scenarios presented in the main text (historical= black; RCP 4.5= yellow; RCP 8.5=red) are depicted relative to scenarios with pathogen development rate *(q)* held constant (historical= gray; RCP 4.5= blue; RCP 8.5=purple).


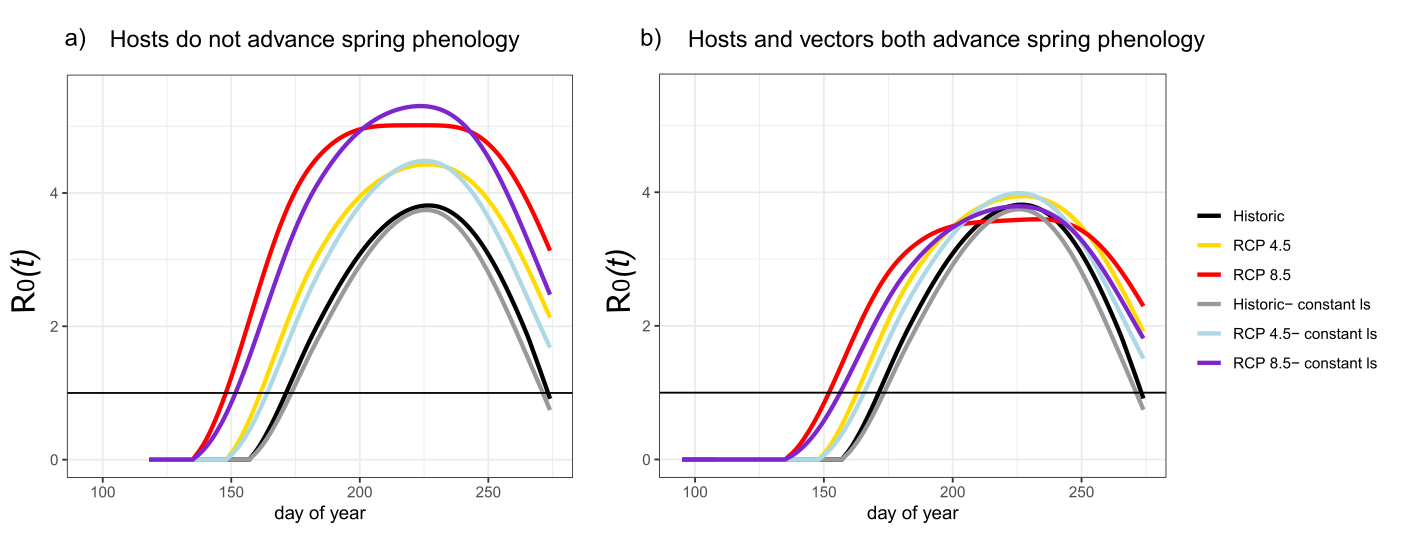
**Figure S4.** Temperature-dependent transmission potential, R_0_, across the breeding season for scenarios in which birds do not arrive earlier under warming (a) and scenarios in which birds advance arrival timing under warming (b). Model scenarios presented in the main text (historical= black; RCP 4.5= yellow; RCP 8.5=red) are depicted relative to scenarios with mosquito lifespan (and mortality, *µ_v_*) held constant (historical= gray; RCP 4.5= blue; RCP 8.5=purple).


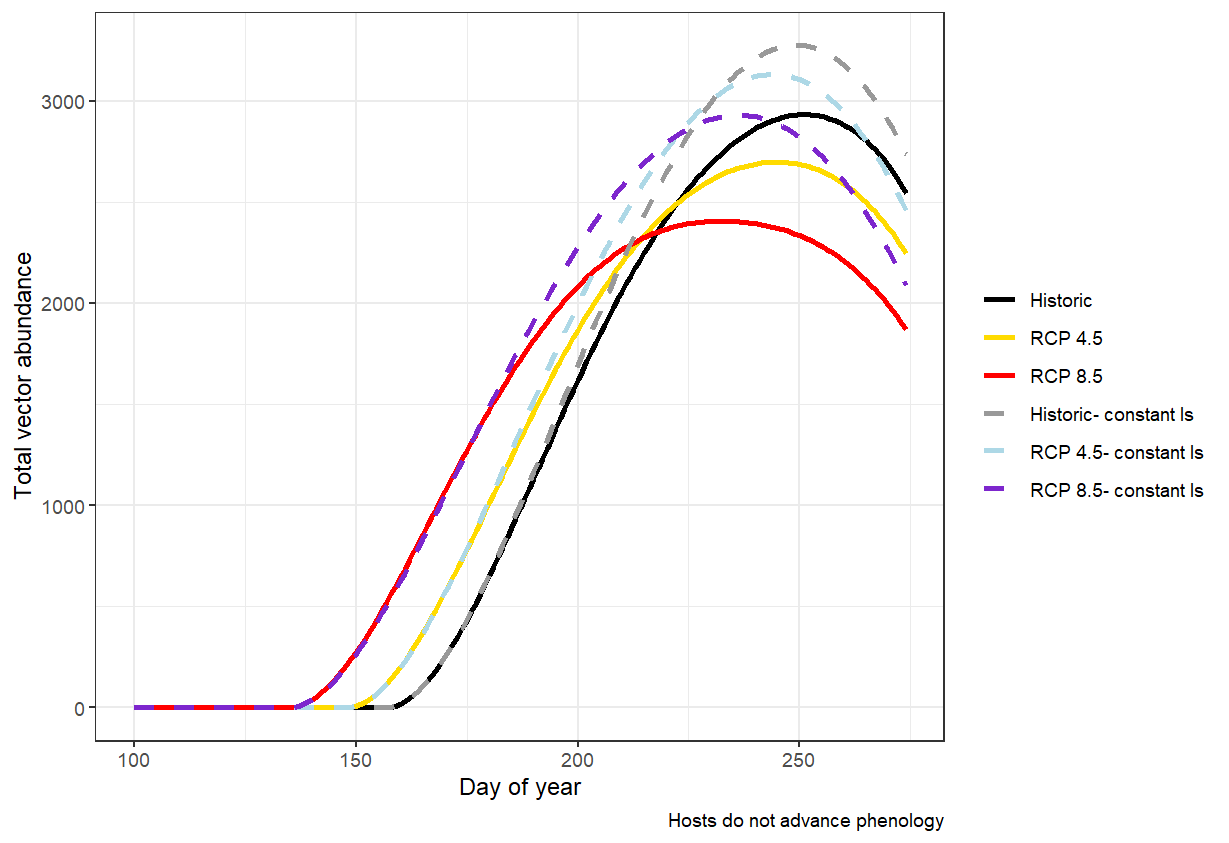


**Figure S5.** Total vector abundance across the breeding season. Model scenarios presented in the main text (solid lines; historical= black; RCP 4.5= yellow; RCP 8.5=orange) are depicted relative to scenarios with mosquito lifespan (and mortality, *µ_v_*) held constant (dashed lines; historical= gray; RCP 4.5= blue; RCP 8.5=purple).

***8. Sensitivity analyses - eliminating the fecundity cost or disease-induced mortality***

In some systems, migratory hosts who do not update spring phenology will not experience a cost to reproduction (e.g. by reducing the time between arrival and nesting (Dunn and Møller, 2014)), and phenological mismatch and lower chick production might not translate to declines in population size (Reed et al., 2013; Iler et al., 2021). To explore the impact of vector-host overlap in the absence of a fecundity cost, we set *c* to zero and re-ran the model. We evaluated how disease-induced impacts on hosts and prevalence dynamics shifted relative to the main model. We found that when there is no fecundity cost, the host to vector ratio is higher and infection spreads more slowly (Figure S6e,f). Given that we still see differences between solid and dashed lines under warming scenarios, changes in vector-host overlap matters independent of impacts on reproduction (Figure S6d-f). The fecundity cost might be important for inferences about timing of peak vector prevalence. In the main model, we saw an earlier peak in the severe warming scenario with no host advancement (Figure S6c) likely due to susceptible host depletion, but the earlier peak did not occur when we set *c* to zero.

We also assessed how eliminating disease-induced mortality impacted model outcomes. We expected that we might see bite dilution and less transmission due to additional recovered hosts in the population. However, we saw that removing disease-induced mortality increased transmission, possibly because infected individuals survived their entire infectious periods, transmitting the virus to more mosquitoes even though there were more hosts overall (Figure S6h-i).


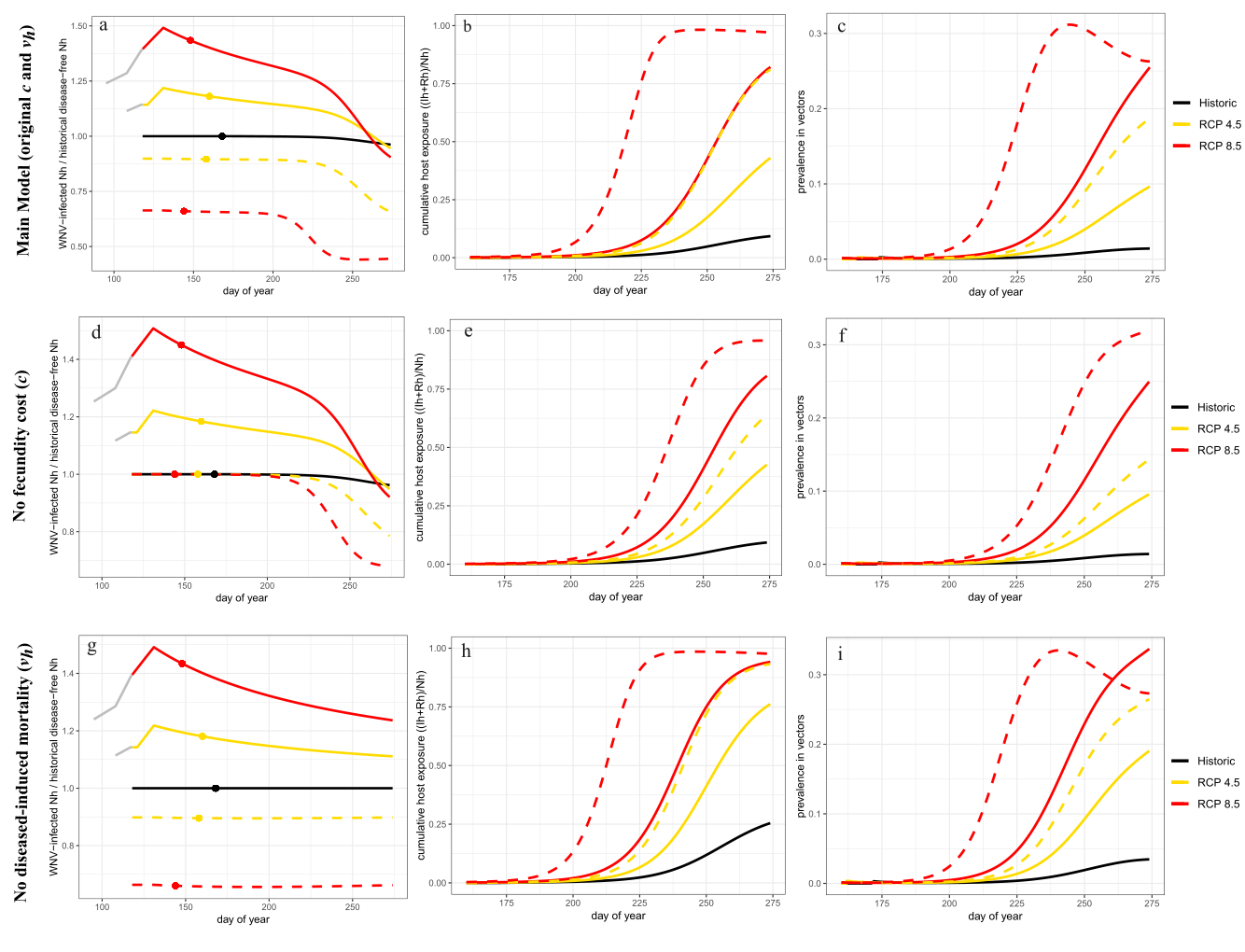


**Figure S6.**  Proportionate decline in host abundance (a,d,g), cumulative host exposure (b,e,h), and vector prevalence (c,f,i) for the main model (top row), the model with no fecundity cost (middle row), and the model with no disease-induced mortality (bottom row). Colors represent the temperature scenarios: black=historical baseline, yellow=RCP 4.5, red=RCP 8.5. Line types represent phenology scenarios: dashed=hosts do not advance arrival timing with warming, solid=hosts arrive earlier under warming.

***9. Sensitivity analyses - breeding season length***

We evaluated model outcomes when the host breeding season window was kept constant under warming. We turned breeding off before *h_end_* in scenarios with earlier host arrival so that in each scenario, the breeding season lasted 156 days. In all scenarios, birds remain present, enabling transmission, until host departure (*h_end_*). We found that qualitative patterns of seasonal prevalence and reductions in host abundance remained the same. One difference was that for all warming scenarios, the end-of-season host abundance post-pathogen introduction was much lower than pre-warming abundance (Figure S7d). Relative to the main model, host and vector infection prevalence reached higher levels when birds arrived earlier under warming (Figure S7e,f). This is likely because, with a fixed breeding season length, host abundances were not as elevated under warming, so the vector to host ratio was higher.


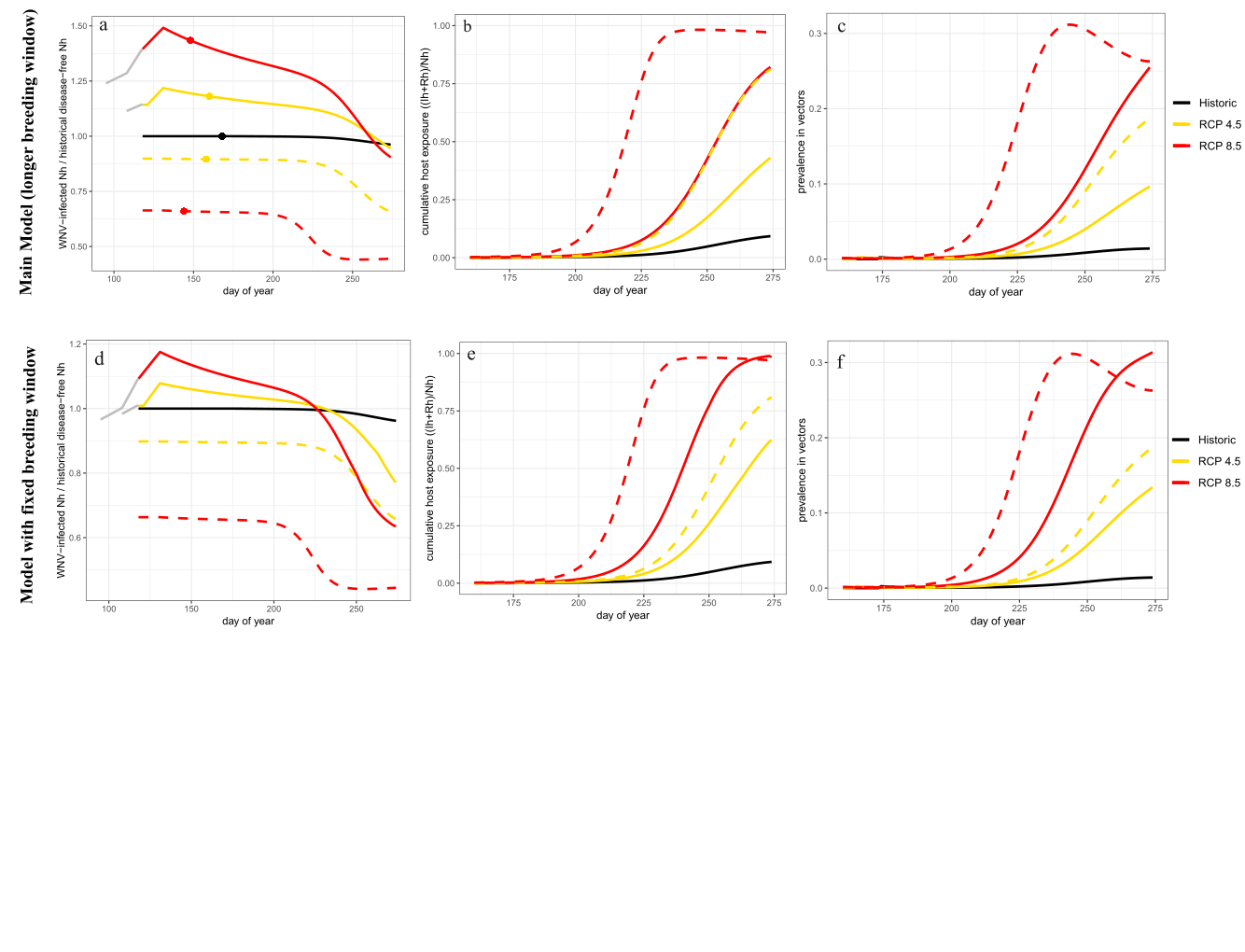


**Figure S7**. Proportionate decline in host abundance (a,d), cumulative host exposure (b,e), and vector prevalence (c,f) for the main model (top row) and the model where breeding season length was held constant (bottom row). Colors represent the temperature scenarios: black=historical baseline, yellow=RCP 4.5, red=RCP 8.5. Line types represent phenology scenarios: dashed=hosts do not advance arrival timing with warming, solid=hosts arrive earlier under warming.

***10. Sensitivity analyses - effects of non-competent hosts in the community***

We explored how the presence of other bird species could impact transmission potential by diluting vector bites on the focal host. We ran the model with *N_c_* equal to 0 (as presented in the main text), 200, and 800. We plotted the peak value of daily R_0_ for each of the 5 scenarios with these three *N_c_* values (Figure S8). Differences between scenarios were dampened as the number of non-competent hosts increased, with no differences between warming scenarios when *N_c_* = 800. Unsurprisingly, transmission potential decreases at higher *N_c_* values, as non-competent hosts are bitten by vectors, but cannot become infected themselves. The patterns presented in the main text (*N_c_* = 0), therefore, are most relevant for a common migratory host preferentially bitten by vectors or a set of ecologically-similar host species responding to an emerging pathogen.


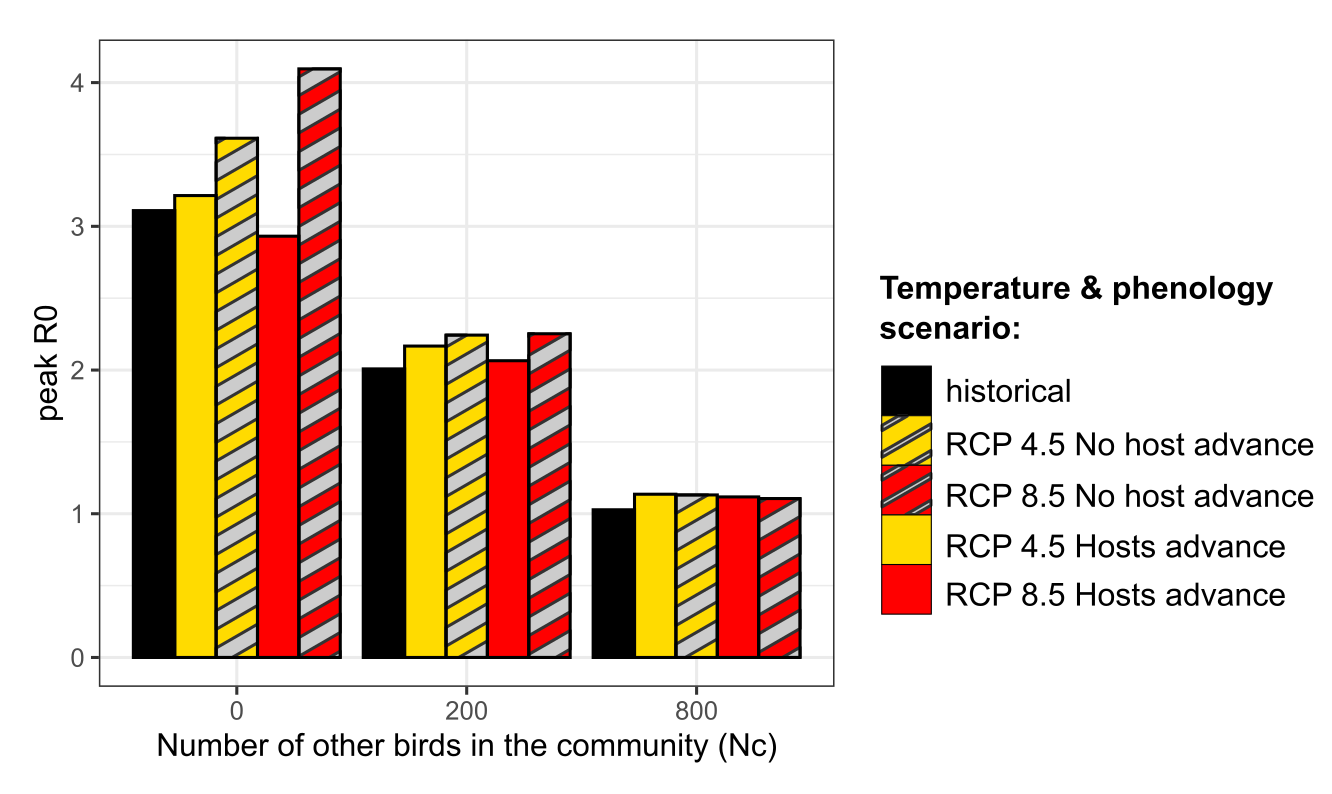


**Figure S8.** Peak daily R_0_ across scenarios for different values of N_C_: 0 (main model value), 200, or 800. Bar colors correspond to the five scenarios (black = historical; solid yellow = RCP 4.5, hosts advance; hatched yellow = RCP 4.5, no host advance; solid red = RCP 8.5, hosts advance; hatched red = RCP 8.5, no host advance).

***References***

Bent, A. C. (1949). *Life histories of North American Thrushes, Kinglets, and their Allies*. Taunton, MA: UNITED STATES GOVERNMENT PRINTING OFFICE.

Bergsman, L. D., Hyman, J. M., and Manore, C. A. (2016). A mathematical model for the spread of west Nile virus in migratory and resident birds. *Mathematical Biosciences and Engineering* 13, 401–424. doi: 10.3934/mbe.2015009

Bolling, B. G., Moore, C. G., Anderson, S. L., Blair, C. D., and Beaty, B. J. (2007). Entomological studies along the Colorado front range during a period of intense West Nile virus activity. *J Am Mosq Control Assoc* 23, 37–46. doi: 10.2987/8756-971X(2007)23[37:ESATCF]2.0.CO;2

Brown, D., and Miller, G. (2016). Band recoveries reveal alternative migration strategies in American Robins. *Animal Migration* 3, 35–47. doi: 10.1515/ami-2016-0004

Brown, L. M., and Hall, R. J. (2018). Consequences of resource supplementation for disease risk in a partially migratory population. *Philosophical Transactions of the Royal Society B: Biological Sciences* 373. doi: 10.1098/rstb.2017.0095

Couper, L. I., Farner, J. E., Caldwell, J. M., Childs, M. L., Harris, M. J., Kirk, D. G., et al. (2021). How will mosquitoes adapt to climate warming? *Elife* 10. doi: 10.7554/eLife.69630

Dahlin, K. J. M., O’Regan, S. M., Han, B. A., Schmidt, J. P., and Drake, J. M. (2024). Impacts of host availability and temperature on mosquito-borne parasite transmission. *Ecol Monogr* 94. doi: 10.1002/ecm.1603

DeSante, D. F., Kaschube, D. R., and Saracco, J. F. (2015). Vital rates of North American landbirds. *The Institute for Bird Populations*.

Diekmann, O., and Heesterbeek, J. A. P. (2000). *Mathematical Epidemiology of Infectious Diseases Model Building, Analysis and Interpretation*. Chichester: John Wiley & Son, Ltd.

Dunn, P. O., and Møller, A. P. (2014). Changes in breeding phenology and population size of birds. *Journal of Animal Ecology* 83, 729–739. doi: 10.1111/1365-2656.12162

Hall, R. J., Brown, L. M., and Altizer, S. (2016). Modeling vector-borne disease risk in migratory animals under climate change. *Integr Comp Biol* 56, 353–364. doi: 10.1093/icb/icw049

Iler, A. M., Caradonna, P. J., Forrest, J. R. K., and Post, E. (2021). Demographic Consequences of Phenological Shifts in Response to Climate Change. *Annu Rev Ecol Evol Syst* 52, 221–245. doi: 10.1146/annurev-ecolsys-011921-032939

Kilpatrick, A. M., Daszak, P., Jones, M. J., Marra, P. P., and Kramer, L. D. (2006). Host heterogeneity dominates West Nile virus transmission. *Proceedings of the Royal Society B: Biological Sciences* 273, 2327–2333. doi: 10.1098/rspb.2006.3575

Komar, N., Langevin, S., Hinten, S., Nemeth, N., Edwards, E., Hettler, D., et al. (2003). Experimental Infection of North American Birds with the New York 1999 Strain of West Nile Virus. *Emerg Infect Dis* 9, 311–322.

LaDeau, S. L., Kilpatrick, A. M., and Marra, P. P. (2007). West Nile virus emergence and large-scale declines of North American bird populations. *Nature* 447, 710–713. doi: 10.1038/nature05829

Laperriere, V., Brugger, K., and Rubel, F. (2011). Simulation of the seasonal cycles of bird, equine and human West Nile virus cases. *Prev Vet Med* 98, 99–110. doi: 10.1016/j.prevetmed.2010.10.013

Murdock, C. C., Foufopoulos, J., and Simon, C. P. (2013). A Transmission Model for the Ecology of an Avian Blood Parasite in a Temperate Ecosystem. *PLoS One* 8, 1–14. doi: 10.1371/journal.pone.0076126

Nowosad, J. (2021). pollen: Analysis of Aerobiological Data. Available at: https://CRAN.R-project.org/package=pollen

Owen, J. C., Landwerlen, H. R., Dupuis, A. P., Belsare, A. V., Sharma, D. B., Wang, S., et al. (2021). Reservoir hosts experiencing food stress alter transmission dynamics for a zoonotic pathogen. *Proceedings of the Royal Society B: Biological Sciences* 288. doi: 10.1098/rspb.2021.0881

Reed, T. E., Jenouvrier, S., and Visser, M. E. (2013). Phenological mismatch strongly affects individual fitness but not population demography in a woodland passerine. *Journal of Animal Ecology* 82, 131–144. doi: 10.1111/j.1365-2656.2012.02020.x

Reisen, W. K. (1995). Effect of Temperature on Culex tarsalis (Diptera: Culicidae) from the Coachella and San Joaquin Valleys of California. *J Med Entomol* 32, 636–645. doi: 10.1093/jmedent/32.5.636

Reisen, W. K., Fang, Y., and Martinez, V. M. (2006). Effects of Temperature on the Transmission of West Nile Virus by Culex tarsalis (Diptera: Culicidae). Available at: https://academic.oup.com/jme/article/43/2/309/1061884

Reisen, W. K., Milby, M. M., Presser, S. B., and Hardy, J. L. (1992). Ecology of Mosquitoes and St. Louis Encephalitis Virus in the Los Angeles Basin of California, 1987–1990. *J Med Entomol* 29, 582–598. doi: 10.1093/jmedent/29.4.582

Rubel, F., Brugger, K., Hantel, M., Chvala-Mannsberger, S., Bakonyi, T., Weissenböck, H., et al. (2008). Explaining Usutu virus dynamics in Austria: Model development and calibration. *Prev Vet Med* 85, 166–186. doi: 10.1016/j.prevetmed.2008.01.006

Saino, N., Ambrosini, R., Rubolini, D., Von Hardenberg, J., Provenzale, A., Hüppop, K., et al. (2011). Climate warming, ecological mismatch at arrival and population decline in migratory birds. *Proceedings of the Royal Society B: Biological Sciences* 278, 835–842. doi: 10.1098/rspb.2010.1778

Shaftel, R., Rinella, D. J., Kwon, E., Brown, S. C., Gates, H. R., Kendall, S., et al. (2021). Predictors of invertebrate biomass and rate of advancement of invertebrate phenology across eight sites in the North American Arctic. *Polar Biol* 44, 237–257. doi: 10.1007/s00300-020-02781-5

Shocket, M. S., Verwillow, A. B., Numazu, M. G., Slamani, H., Cohen, J. M., Moustaid, F. El, et al. (2020). Transmission of West Nile and five other temperate mosquito-borne viruses peaks at temperatures between 23˚C and 26˚C. *Elife* 9, 1–67. doi: 10.1101/597898

Stewart, R. L. M., Francis, C. M., and Massey, C. (2002). Age-related differential timing of spring migration within sexes in passerines. *Wilson Bulletin* 114, 264–271. doi: 10.1676/0043-5643(2002)114[0264:ARDTOS]2.0.CO;2

Tøttrup, A. P., and Thorup, K. (2008). Sex-differentiated migration patterns, protandry and phenology in North European songbird populations. *J Ornithol* 149, 161–167. doi: 10.1007/s10336-007-0254-x

Vanderhoff, N., Pyle, P., Patten, M. A., Sallabanks, R., and James F. C. (2016). American Robin (Turdus migratorius). *The Birds of North America*.

Wonham, M. J., Lewis, M. A., Rencławowicz, J., and Van Den Driessche, P. (2006). Transmission assumptions generate conflicting predictions in host-vector disease models: A case study in West Nile virus. *Ecol Lett* 9, 706–725. doi: 10.1111/j.1461-0248.2006.00912.x

Youngflesh, C., Montgomery, G. A., Saracco, J. F., Miller, D. A. W., Guralnick, R. P., Hurlbert, A. H., et al. (2023). Demographic consequences of phenological asynchrony for North American songbirds. *Proceedings of the National Academy of Sciences* 120. doi: 10.1073/pnas.2221961120
